# Visualizing the complex functions and mechanisms of the anaphase promoting complex/cyclosome (APC/C)

**DOI:** 10.1098/rsob.170204

**Published:** 2017-11-22

**Authors:** Claudio Alfieri, Suyang Zhang, David Barford

**Affiliations:** MRC Laboratory of Molecular Biology, Francis Crick Avenue, Cambridge CB2 0QH, UK

**Keywords:** APC/C, cell cycle, spindle assembly checkpoint, cryo-EM

## Abstract

The anaphase promoting complex or cyclosome (APC/C) is a large multi-subunit E3 ubiquitin ligase that orchestrates cell cycle progression by mediating the degradation of important cell cycle regulators. During the two decades since its discovery, much has been learnt concerning its role in recognizing and ubiquitinating specific proteins in a cell-cycle-dependent manner, the mechanisms governing substrate specificity, the catalytic process of assembling polyubiquitin chains on its target proteins, and its regulation by phosphorylation and the spindle assembly checkpoint. The past few years have witnessed significant progress in understanding the quantitative mechanisms underlying these varied APC/C functions. This review integrates the overall functions and properties of the APC/C with mechanistic insights gained from recent cryo-electron microscopy (cryo-EM) studies of reconstituted human APC/C complexes.

## 1. The anaphase promoting complex or cyclosome regulates cell cycle transitions

The anaphase promoting complex or cyclosome (APC/C) is a multi-subunit cullin-RING E3 ubiquitin ligase that functions to regulate progression through the mitotic phase of the cell cycle and to control entry into S phase [1–4]. The APC/C also plays a role in regulating meiosis, and has been implicated in post-mitotic functions including dendrite formation in neurons, as well as metabolic, learning and memory processes [5–10]. APC/C-mediated coordination of cell cycle progression is achieved through the temporal and spatial regulation of APC/C activity and substrate specificity. The APC/C becomes activated at the onset of mitosis, and ubiquitinates Nek2A and cyclin A (an S- and M-phase cyclin) at prometaphase. At metaphase, the APC/C targets for degradation two inhibitors of the anaphase transition, namely, securin and cyclin B (M-phase cyclin) [11,12]. Securin is a protein inhibitor of separase, a protease that cleaves the cohesin subunit kleisin [13]. Cleavage of kleisin disassembles cohesin to trigger sister chromatid segregation and the onset of anaphase [14–16], reviewed in Nasmyth [17]. Reduced cyclin B levels are also required for entry into anaphase, since Cdk1 (cyclin-dependent kinase 1)-cyclin B1 inhibits separase [18–20]. After anaphase, cyclin destruction continues to maintain negligible Cdk activity, necessary for the cell to disassemble the mitotic spindle and exit mitosis [12,21–25]. During G1, the main role of the APC/C is to sustain low levels of mitotic Cdk activity to allow for resetting of replication origins as a prelude to a new round of DNA replication in S phase [26,27].

The temporal regulation of APC/C activity is achieved through a combination of two structurally related coactivator subunits, Cdc20 and Cdh1 [28–38], coupled to protein phosphorylation, APC/C inhibitors and differential affinity for APC/C substrates. The two APC/C coactivators have opposing activity profiles. Cdc20 activates the APC/C during early mitosis when the APC/C is phosphorylated and Cdh1 activity is low due to its Cdk-dependent phosphorylation, whereas APC/C^Cdc20^-mediated reduction of Cdk activity stimulates Cdh1. In turn, APC/C^Cdh1^ ubiquitinates Cdc20, leading to APC/C^Cdc20^ inactivation (with Cdc20 auto-ubiquitination also playing a role [39]). Thus, Cdc20 activates Cdh1 that in turn antagonizes Cdc20 activity. The switching between APC/C^Cdc20^ and APC/C^Cdh1^ fulfils two main functions. First, APC/C^Cdc20^ and APC/C^Cdh1^ have over-lapping but nevertheless distinct substrate specificities. Therefore, specific cell cycle regulators are degraded during the separate phases of APC/C^Cdc20^ and APC/C^Cdh1^ activity, allowing for ordered progression through the cell cycle. Second, Cdc20 and Cdh1 are subject to control by different regulatory mechanisms. Cdc20 activates the APC/C that is phosphorylated by Cdk and Plk1 protein kinases during early mitosis, whereas Cdh1 is inhibited by its Cdk-mediated phosphorylation. Importantly, APC/C^Cdc20^ activity towards securin and cyclin B is inhibited by the mitotic checkpoint complex (MCC), a multi-protein complex activated by the spindle assembly checkpoint (SAC), reviewed in Lara-Gonzalez *et al.* [40] and Musacchio [41]. The SAC ensures that anaphase is delayed until every chromosome is aligned on the mitotic spindle. Emi1 inhibits metazoan APC/C^Cdh1^ during interphase [42–44], whereas Acm1 inhibits *Saccharomyces cerevisiae* APC/C^Cdh1^ [45,46]. The structurally related protein Emi2 (XErp1) regulates the APC/C in embryonic cells and meiosis [47].

## 2. The APC/C is a multi-subunit cullin-RING E3 ligase

The large size and complex architecture of the APC/C is intimately linked to its regulatory mechanisms involving control by reversible phosphorylation, the SAC, Emi1 and interchangeable coactivator subunits. These regulatory mechanisms ensure the APC/C is controlled in a cell-cycle-dependent manner and that its substrate specificity is also modulated throughout the cell cycle.

*Subunit composition.* The APC/C comprises the core complex (14 subunits in metazoans, 13 in yeast) [48–59], together with the interchangeable coactivator subunits (either Cdc20 or Cdh1) [28,29,31] (table 1). APC/C subunits are functionally and structurally organized into three classes: the catalytic module, the substrate recognition module and the scaffolding module (table 1). The catalytic module comprises Apc11, the RING domain subunit [61–63] and Apc2, the cullin subunit [50,51,63]. These two subunits are orthologues of Rbx1 and the cullin subunit of cullin-RING ligases (CRLs), respectively. In both the APC/C and CRLs, an N-terminal β-strand of the RING domain subunit is integrated within the β-sheet of the C-terminal domain (CTD) of the cullin subunit. As discussed below, the catalytic module incorporates two conformationally-variable domains, the RING domain of Apc11 (Apc11^RING^) and the WHB domain of Apc2 (Apc2^WHB^), both attached to the CTD of Apc2 (Apc2^CTD^) by flexible linkers. The conformational flexibilities of Apc2^WHB^ and Apc11^RING^ have important implications for APC/C catalysis and regulation.

**Table 1.** Subunits of the human anaphase promoting complex/cyclosome (APC/C). Alternative *S. cerevisiae* subunits in parenthesis.

subunit	length (aa)	stoichiometry	location	domain/Region 1	domain/Region 2	domain/Region 3	phosphorylation sites (from ref. [60])
Apc1	1944	1	scaffolding module platform	WD40 domain (1–612)	mid-N (613–986) mid-C (1617–1944)	PC domain (1013–1616)	60,65,202,233,286,291,297,298,299,309,313,316,317,341,343,351,355,362,364,372,373,377,386,389,394,416,501,518,520,522,524,530,536,537, 542,547,555,563,564,569,576,582,600,686,688,699,701,703,731,916,920,921,922,1001,1347,1349
Apc2	822	1	catalytic module	NTD (1–432) cullin repeats	CTD (433–822) including WHB domain	—	205,218,314,466,470,474,532,534,732,736,738,742
Apc3A Apc3B (Cdc27)	824	2	scaffolding module TPR lobe	TPR dimer interface TPR motifs 1–7 (1–535)	TPR superhelix TPR motifs 8–14 (536–824)	—	183,185,186,192,194,200,203,205,209,219,220,222,225,228,230,231,233,237,241,244,251,252,255,264,267,276,279,281,289,291,302,304,312, 313,327,329,331,334,336,343,349,351,352,356,357,358,364,366,368,369,383,384,386,387,388,389,419,426,430,434,435,438,443,444,446, 761,800,803,806,807,809,814,821
Apc4	808	1	scaffolding module platform	WD40 domain/4HBD	—	—	199,469,488,757,758,777,779
Apc5	755	1	scaffolding module platform	NTD (1–169)	TPR superhelix TPR motifs 1–13 (206–755)	—	15,130,178,179,195,221,228,232,674
Apc6A Apc6B (Cdc16)	620	2	scaffolding module TPR lobe	TPR dimer interface TPR motifs 1–7 (1–261)	TPR superhelix TPR motifs 8–14 (262–620)	—	112,559,573,577,580,584,585,592,599,607,614
Apc7A Apc7B	599	2	scaffolding module TPR lobe	TPR dimer interface TPR motifs 1–3 (21–166)	TPR dimer interface TPR motifs 4–7 (167–359)	TPR superhelix TPR motifs 8–14 (360–599)	119,120,123,125,126,573,582,584
Apc8A Apc8B (Cdc23)	597	2	scaffolding module TPR lobe	TPR dimer interface TPR motifs 1–7 (1–287)	TPR superhelix TPR motifs 8–14 (288–597)	—	562,565,582,584,588,593,596
Apc10	185	1	degron recognition module	Doc homology (2–162)	IR tail (163–185)	—	—
Apc11	84	1	catalytic module	β-strand (1–18)	RING domain (21–84)	—	—
Apc12A Apc12B (Cdc26)	85	2	scaffolding module TPR lobe	N-term (1–25) Extended chain, short α-helix	—	—	42,51,52,56,78
Apc13 (Swm1)	74	1	scaffolding module TPR lobe	extended chain (1–67)	—	—	
Apc15 (Mnd2)	121	1	scaffolding module platform	extended chain and α-helix (1–56)		—	76,80,98
Apc16	110	1	scaffolding module TPR lobe	α-helix (52–110)	—	—	8,16,26
Cdc20/ Cdh1	499/496	1	degron recognition module	NTD (73–135/42–163)	WD40 domain (168–471/172–473)	IR tail (492–499/483–496)	—
UbcH10	179	1	catalytic module	UBC domain (30–179)	—	—	—

Together, the coactivators and Apc10 form the substrate recognition module, with the coactivator's WD40 β-propeller domain being primarily responsible for mediating degron recognition (D box, KEN box and ABBA motif) [64–71]. Optimal D-box recognition requires the core APC/C subunit Apc10 (Doc1 in *S. cerevisiae*) [54,72,73]. The substrate recognition and catalytic modules represent the key functional subunits of the APC/C, reflected in their high degree of conservation. It is striking that these two functional modules represent only 15% of the total mass of the molecule. Most of the APC/C mass is conferred by the seven large scaffolding subunits, four of which form homo-dimers—further contributing to the high relative mass of the scaffolding module [74]. Remarkably, the majority of APC/C subunits, particularly the scaffolding subunits, are composed of multiple repeat motifs. Five scaffolding proteins are tetratricopeptide repeat (TPR) proteins, being composed of 13–14 TPR motifs arranged in contiguous arrays. TPR proteins, ubiquitous in all three domains of life, were first discovered in what were later identified as yeast APC/C subunits [75–78]. Their presence in multiple protein complexes of diverse functions such as the APC/C indicates a role in mediating protein–protein interactions and the assembly of multi-protein complexes [79]. Later, atomic resolution structural analysis of the APC/C provided a mechanistic rationale for many of the previously characterized TPR mutations [80–83].

The four canonical TPR proteins (Apc3, Apc6, Apc7, Apc8) are structurally highly homologous, being composed almost entirely of 14 TPR motifs. These self associate to form homo-dimers [81–83]. Apc1, the largest APC/C subunit, features another type of motif that is only observed in Apc1 and the Rpn1 and Rpn2 subunits of the 19S regulatory subunit of the proteasome (in exactly the same number and arrangement) [84]. These approximately 40-residue motifs are termed the PC (proteasome-cyclosome) repeat [85]. Although not discernable in sequence, cryo-electron microscopy (cryo-EM) studies revealed that Apc1 contains an N-terminal seven-bladed β-propeller domain [80,86]. Apc4 also comprises a β-propeller domain [87]. Finally, four small intrinsically disordered subunits (vertebrate Apc12, Apc13, Apc15, Apc16) function as TPR-accessory subunits. These subunits interact with TPR subunits and, as explained later, Apc12, Apc13 and Apc16 stabilize TPR subunits and mediate inter-TPR interactions [51,54,56,80,86,88,89]. Apc15 is not required for APC/C assembly. It functions to negatively regulate the SAC by controlling the stability of the Cdc20 subunit of the MCC through APC/C-dependent auto-ubiquitination [59,90–95].

Structural investigations of the APC/C were initiated some 18 years ago, shortly after its discovery in 1995 [1–3]. Initial efforts focused on a complementary approach of crystallography of individual APC/C subunits and small sub-complexes and homologous proteins [71,81–84,87,89,96–100], together with single particle cryo-EM studies of the intact complex that represented various functional states of the complex purified from endogenous sources: budding yeast, fission yeast, *Xenopus* and human [73,101–107]. A combination of crystallography of individual APC/C subunits, native mass spectrometry [74] and electron microscopy provided information on the subunit stoichiometry of the APC/C (table 1).

The recent progress in understanding the structure and mechanisms of the APC/C through atomic resolution structures of various functional states of the complex resulted from technical developments in reconstituting the recombinant APC/C [74,91,108] together with recent advances in single particle cryo-electron microscopy (direct electron detectors and software for image analysis and 3D-reconstructions) [109]. Recent EM studies have focused on reconstituted human APC/C complexes [80,86,92,93,99,110–112].

In 2014 a 7.4 Å resolution structure of the reconstituted APC/C^Cdh1.substrate^ complex was published [86]. At 7.4 Å resolution the secondary structural architecture can be defined. Alpha-helices are resolved as rod-like structures, whereas β-sheets are visualized as planar structures. The subunit assignment of the electron microscopy (EM) density map was determined based on two approaches. One was a subunit deletion approach where the structures of reconstituted APC/C complexes lacking defined subunits were compared with the wild-type complex [74]. Difference density due to the deleted subunit could be assigned to a specific subunit. In a related approach, comparing two complexes that share a common subunit allows its identification. However Apc1, an essential subunit required for APC/C stability, which therefore cannot be deleted without disrupting the entire complex, was identified based on a process of elimination and by recognizing architectural features of the PC domain in the EM density map [80,86]. Finally, Apc13 in *S. cerevisiae* was identified through locating GFP fused to its C-terminus [74]. Importantly, EM density for Apc2^CTD^ was weak and diffuse whereas that for Apc11^RING^ and Apc2^WHB^ was not visible, indicating a high degree of conformational flexibility of the catalytic module. Conformational heterogeneity was also confirmed through 3D classification of the cryo-EM dataset [86]. Altogether, the EM studies revealed a striking degree of structural conservation from yeast to metazoan. The APC/C of higher eukaryotes differs from yeast because of an additional TPR subunit (Apc7) situated on the top of the TPR lobe that interacts only with Apc3 (table 1). The role of Apc7 has yet to be defined.

The 7.4 Å resolution structure of the APC/C was soon followed by a near-atomic resolution structure of the complex of APC/C^Cdh1^ with the inhibitor Emi1 (APC/C^Cdh1.Emi1^) [80]. This structure was at 3.6 Å resolution overall, but a local resolution map showed that the more rigid regions of the map were closer to 3.2 Å resolution. Two regions in particular were recovered at lower resolution (approx. 5 Å) due to their higher relative flexibility. These were the catalytic module formed of Apc11 and Apc2^CTD^, and the coactivator Cdh1.

The 3.6 Å resolution cryo-EM map of APC/C^Cdh1.Emi1^ provided the basis for understanding the detailed architecture of the APC/C and served as a template for understanding subsequent different functional states, some at lower resolution. Building of the atomic-resolution model was based on fitting of atomic coordinates of X-ray structures of most of the large subunits and close homologues. For Apc1, fitting to the N-terminal WD40 domain and densities adjacent to its central PC domain (Apc1^PC^) that lack structural homologues was performed *ab initio*. The TPR accessory subunits Apc13, Apc15 and Apc16 were also built *ab initio* [80].

**Figure 1.**
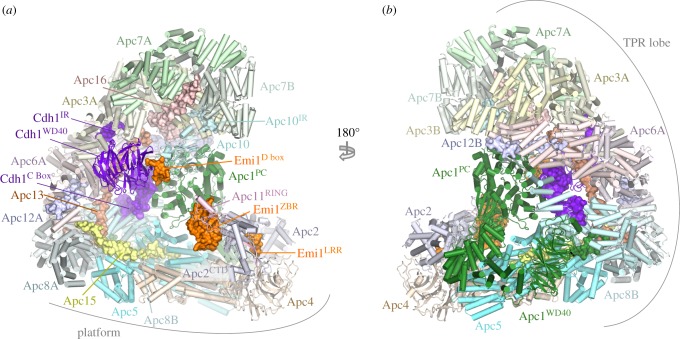
Overall structure of the human APC/C^Cdh1.Emi1^ complex. (*a*) and (*b*) Two orthogonal views of the APC/C. Large APC/C subunits are represented as cartoons, whereas small APC/C subunits (Apc12, Apc13, Apc15, Apc16), the IR tails of Cdh1 and Apc10, the Cdh1 NTD and the Emi1 inhibitor are shown as space filling representations. The TPR and platform sub-structures are labelled. The two subunits of the canonical homo-dimeric TPR subunits (Apc3, Apc6, Apc7 and Apc8) and Apc12 are labelled with the suffix ‘A’ and ‘B’. Apc2^CTD^ and Apc11^RING^ form the catalytic module, Cdh1 and Apc10 generate the substrate recognition module. PDB 4UI9, from Chang *et al.* [80].

The APC/C adopts a triangular shape delineated by a lattice-like shell organized into two sub-structures (figure 1) [80,86]. The back and top of the complex is formed from a bowl-shaped TPR lobe, an assembly of the four canonical TPR proteins (Apc3, Apc6, Apc7, Apc8) and three TPR accessory subunits (table 1). The base of the APC/C comprises the platform subunits Apc4 and Apc5, together with two (non-PC) domains of Apc1. Apc1^PC^ extends from the platform to contact the TPR lobe. Together, the TPR lobe and platform sub-structures define a central cavity. The degron recognition module of coactivator and Apc10 is located at the top of the cavity with Apc10 interacting extensively with Apc1^PC^. The catalytic module of Apc2-Apc11 is positioned at the periphery of the platform such that Apc2^CTD^ and associated Apc11 are at the front of the cavity situated directly below Apc10 and Cdh1.

**Figure 2.**
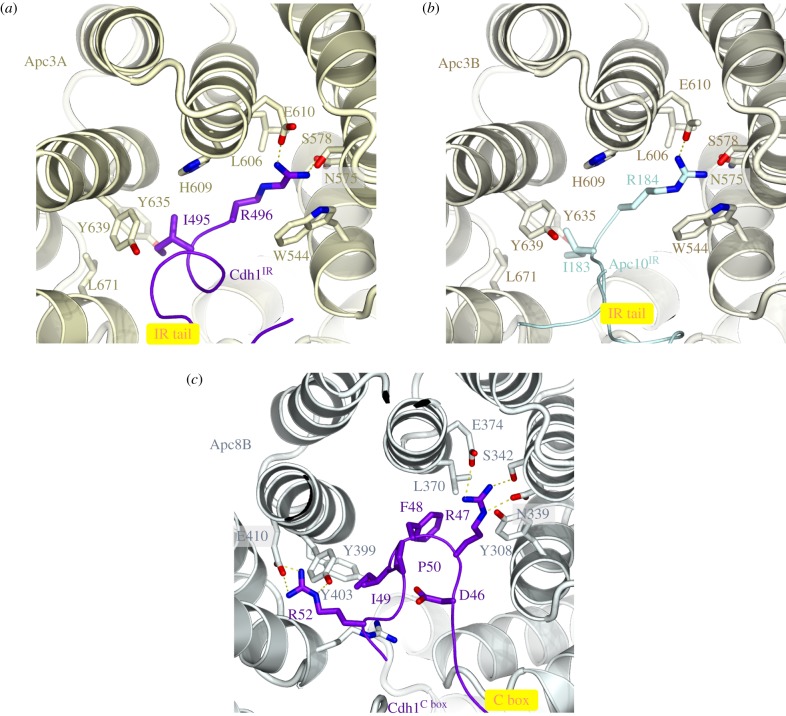
The IR-tail and C-box binding sites of Apc3 and Apc8 respectively, are homologous. (*a*) Cdh1 IR-tail binding site. (*b*) Apc10 IR-tail binding site. (*c*) C-box binding site on Apc8B. The Ile and Arg side chains of the IR tail of both Cdh1 and Apc10 interact with a site on Apc3 that is homologous to the binding sites for Arg(47) and Ile(49) of the Cdh1 C box on Apc8B. The C box (DR[F/Y]IPxR) forms additional contacts to Apc8B as shown. PDB 4UI9, from Chang *et al.* [80].

The canonical TPR proteins form structurally related V-shaped homo-dimers [81–83]. Each subunit comprises an α-helical solenoid with two turns of TPR helix. Whereas the N-terminal TPR helix forms the homo-dimer interface, the C-terminal TPR helix creates a protein-binding groove. Apc6 binds its accessory subunit Apc12 through this groove (figure 1) [82,89], stabilizing Apc6 [89], whereas the Apc3 and Apc8 homo-dimers use one of their dyad-related C-terminal grooves to engage the coactivator subunits (either Cdc20 or Cdh1) (figures 1 and 2) [60,80,86]. Within the TPR lobe, the four canonical TPR proteins stack in a parallel array generating a left-handed super-helix that adopts pseudo dyad-symmetry. Together the TPR accessory subunits Apc13 and Apc16 (and presumably Apc9 in *S. cerevisiae*) interact with structurally and symmetry related sites on seven of the eight TPR subunits to stabilize the TPR lobe and contribute to defining the order of TPR protein assembly [80].

**Figure 3.**
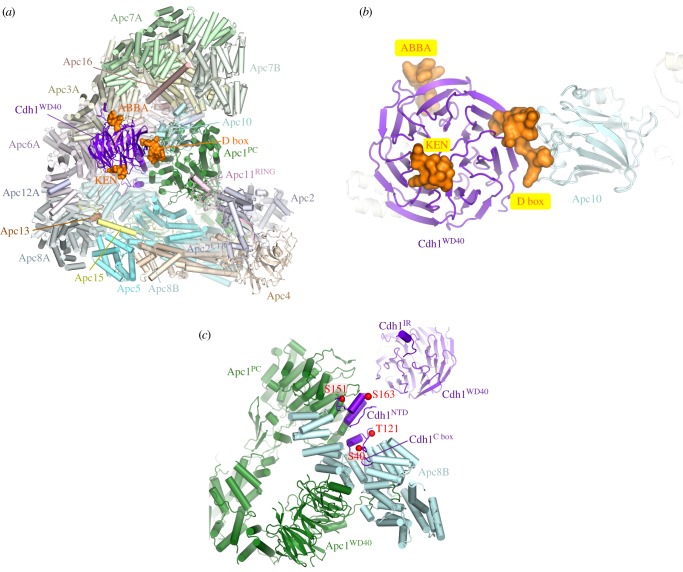
Coactivators interact with Apc1 and Apc3 and create a D-box co-receptor with Apc10. (*a*) Overview of the APC/C with the Cdh1 coactivator subunit. Based on the APC/C^Cdh1.Emi1^ coordinates (PDB 4UI9) [80] with the KEN box and ABBA motif modelled on the *S. cerevisiae* Cdh1–Acm1 complex (PDB: 4BH6) [71]. Except for the D box, Emi1 coordinates are not shown. (*b*) Close-up view of the D-box co-receptor formed from Cdh1 and Apc10. (*c*) Cdk1-dependent phosphorylation of the NTD of Cdh1 blocks its binding to the APC/C. Red spheres indicate sites of inhibitory phosphorylation.

Apc10 and both coactivators share structurally related C-terminal Ile-Arg motifs (IR tails) that interact with the C-terminal TPR motifs of Apc3 (figures 1 and 2*a*,*b*) [66,88,96,113,114]. Additionally, coactivators comprise a C-box motif within their N-terminal domain (NTD) [68] that mediates interactions with the APC/C [68,113], dependent on Apc8 [115]. Due to the presence of multiple binding sites on the TPR lobe, the pseudo dyad-symmetry of the TPR lobe has important consequences for mechanisms of interaction with coactivators and substrates. Not only does the dyad symmetry of each TPR protein mean that there is multiplication of protein/ligand binding sites (for example the common IR tails of coactivator and Apc10 interact with separate subunits of the Apc3 homo-dimer (figure 2*a*,*b*)), but also the IR-tail binding site on Apc3 is structurally related to the C-box binding site on Apc8B, a paralogue of Apc3 (figure 2*c*). The mechanism of interaction of the IR tail with Apc3 is structurally analogous to that of the R[F/Y]I motif of the C box with the C-box binding site on Apc8B [80]. Because of this, the structurally equivalent C-box binding site on Apc8A is capable of binding the IR tail of Cdc20^MCC^ (in the APC/C^MCC^ complex) [92,93]. A conformational transition involving the C-terminal TPR motifs of Apc3A occludes the coactivator IR-tail binding pocket in the absence of the IR-tail ligand [60,80,100]. Finally, regions of the NTD of coactivator also interact with Apc1^PC^ (figure 3*c*). Thus the degron-recognition WD40 domains of the coactivators are connected to the APC/C scaffold through three sites, attached through flexible linkers. This allows for conformational flexibility of the WD40 domain.

In the platform, analogous to the Apc6–Apc12 interaction, the C-terminus of Apc15 inserts into the TPR groove of Apc5 as an extended chain, with its N-terminal α-helix (Apc15^NTH^) bridging Apc5 and Apc8 [80].

## 3. Coactivators are primarily responsible for degron recognition

**Figure 4.**
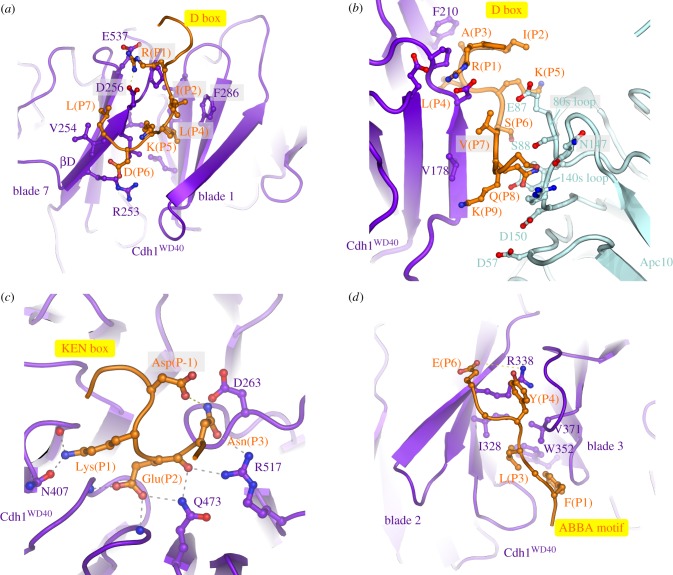
Substrate recognition is mediated by coactivators and Apc10. (*a*) D-box receptor on Cdh1, (*b*) D-box co-receptor (Cdh1 and Apc10), (*c*) KEN-box receptor on Cdh1, (*d*) ABBA-motif interactions with Cdh1. Coordinates in (*a*,*c*,*d*) are based on the *S. cerevisiae* Cdh1–Acm1 complex (PDB: 4BH6) [71]. (*b*) Based on APC/C^Cdh1.Emi1^ complex (PDB 4UI9) [80].

**Figure 5.**
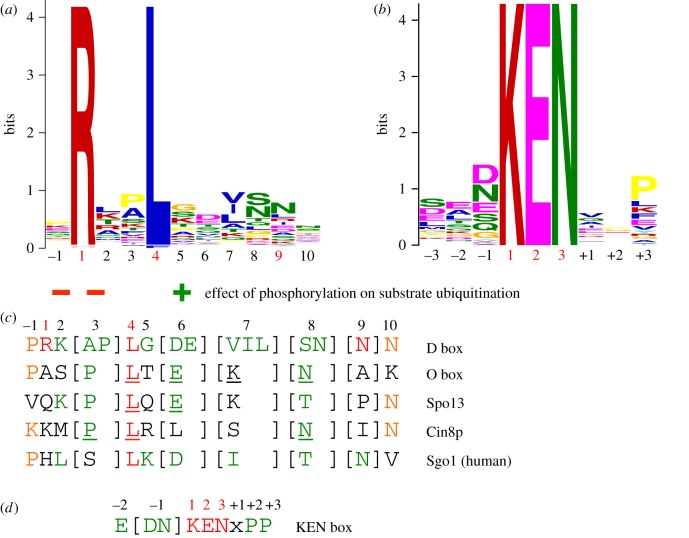
Degron consensus sequences. (*a*) Sequence motif of D box derived from 68 APC/C substrates [71]. Sequence motif determined using multiple expectation maximization for motif elicitation (MEME) [116]. (*b*) Sequence motif of KEN box derived from 46 APC/C substrates [71]. (*c*) Alignment of consensus D box degron with non-canonical D box degrons. (*d*) Consensus KEN box. Adapted from [71].

The APC/C recognizes and ubiquitinates a variety of cell cycle substrates in a cell-cycle-dependent manner. Selection of substrates in a temporal manner is dependent on a variety of factors, but critical among these is the role of coactivators [29]. The APC/C is inactive without coactivator. One function of coactivators is to provide degron recognition sites that engage degrons present in most APC/C substrates [66,69–71], thereby recruiting substrates to the APC/C (figures 1, 3*a*,*b*, 4 and 5). In a few exceptions, for example Nek2A, the core APC/C recognizes substrates, bypassing degron recognition sites on the coactivator. However, Nek2A ubiquitination still relies upon the coactivator-induced stimulation of UbcH10-binding to the APC/C [86,117].

Due to the critical role coactivators play in defining APC/C activity, regulation of APC/C activity by phosphorylation and inhibitory complexes such as the MCC, Emi1 and Acm1 is exerted primarily at the level of coactivators, either by controlling their interaction with the APC/C or by controlling coactivator interaction with degrons. Cdc20 activates the APC/C from early mitosis to anaphase after which Cdh1 binds to the APC/C through to late G1. Switching of these two highly structurally conserved and related coactivators at anaphase changes the substrate specificity and regulatory properties of the APC/C. Cdc20 is thought to recognize a restricted set of substrates (specifically cyclin A, cyclin B and securin), whereas Cdh1 is proposed to have a broader substrate specificity, being able to ubiquitinate all Cdc20 substrates, and in addition recognizes the Aurora A and B kinases, which are not substrates of APC/C^Cdc20^ [118]. Aurora kinases are recognized by APC/C^Cdh1^ through their essential N-terminal A box motif [119]. The role of the C-terminal D box of Aurora kinases is disputed, as discussed in Davey & Morgan [120]. Both coactivators mediate interactions of substrates harbouring D-box and KEN-box motifs to the APC/C. Optimal interactions of the D box also require the Apc10 subunit [54,72,73]. The ABBA motif is recognized by vertebrate Cdc20 [121], and *S. cerevisiae* Cdh1 [71] and Cdc20 [120,122]. In *S. cerevisiae* a specific coactivator termed Ama1 controls meiosis [123,124] that in turn is antagonized by the Mnd2 (Apc15) subunit [125,126].

To understand structurally how coactivators recognize D-box and KEN-box substrates, advantage was made of the fact that many APC/C inhibitors incorporate pseudo-substrate motifs that mimic D-box and KEN-box degrons in order to block substrate recognition. These inhibitors interact with higher affinity with coactivators than do substrates, thereby facilitating the biochemical isolation and crystallization of these complexes. A structure of the MCC from *Schizosaccharomyces pombe*, a complex of Cdc20, Mad2 and BubR1/Mad3, revealed how a KEN box and D box present in BubR1/Mad3 interact with their respective binding sites on the β-propeller domain of Cdc20 [69]. These findings were confirmed and extended in a structure of the β-propeller domain of Cdh1 in complex with Acm1, a Cdh1 specific inhibitor from *S. cerevisiae* [71]. The latter structure also revealed how the ABBA motif (A motif in Acm1 terminology [127]) interacts with Cdh1 (figures 3 and 4). A further study in which human Cdc20 was crystallized with a peptide modelled on the BubR1 KEN box also revealed details of Cdc20 interactions with the KEN-box motif [70].

*D box.* The classical APC/C degron is the destruction box or D box, a ten-residue motif (RxxLx[D/E][Ø]xN[N/S]) (figure 5*a*,*c*) first characterized in B-type cyclins as being necessary and sufficient for APC/C mediated ubiquitination [128–130]. Mutation of any of the three most highly conserved residues, Arg (P1), Leu (P4) or Asn (P9), ablated the destruction signal [128]. The D box binds in a mainly extended conformation to a shallow groove at the side of the β-propeller, found between the two β-blades 1 and 7 (figures 3*b* and 4*a*). The essential Leu (P4) residue anchors the D box to the channel within a hydrophobic pocket, whereas the N-terminal Arg (P1) residue interacts with an acidic pocket at the N-terminus of the channel (figure 4*a*). A conserved acidic residue at P6 interacts with an invariant Arg, whereas a hydrophobic residue at P7, conserved in many D-box motifs, interacts with a hydrophobic surface of the β-propeller (figures 4*a* and 5*a*,*c*) [69,71]. The side chain of P3 abuts a conserved Phe of the coactivator, likely accounting for the high occurrence of residues with small unbranched side chains at this D-box position (figures 4*a* and 5*a*,*c*). Although Arg and Leu are strongly preferred at P1 and P4, respectively, even these two residues are not strictly necessary. For example, in *Drosophila melanogaster* cyclin A [131] and *Homo sapiens* cyclin B3 [132], Phe is substituted for Leu.

Significantly, the conserved C-terminal hydrophilic residues (P8 to P10) do not interact with the coactivator, however the cryo-EM structure of APC/C^Cdh1.Emi1^ (where the inhibitor Emi1 incorporates a D box) showed clear EM density extending from the P7 residue of the D box (interacting with the D-box site on Cdh1) to Apc10 [80]. This showed that the C-terminus of the D box interacts with a hydrophilic surface of Apc10 [80,96,97] involving polar and charged residues on two surface-exposed loops (the 80s and 140s loops) (figure 4*b*). This highly conserved region is required for D-box-dependent APC/C E3 ligase activity, and this potentially dynamic hydrophilic surface may allow for the accommodation of a variety of small polar residues at D-box positions P8 to P10 (figure 5*a*). Disruption of the 140s loop impairs D-box-dependent substrate recognition [133] and Ala substitutions of Ser88 and Asn147 of Apc10 attenuated APC/C^Cdh1^ activity [80]. Although Apc10 primarily interacts with D-box residues P8 to P10, its 80s loop also contacts N-terminal residues of the D box (figure 4*b*). For example, the side chain of Glu87 (invariant in Apc10 orthologues) is sandwiched between P2, P5 and P7 of the D box, perhaps explaining the occurrence of basic residues at these positions, especially for the non-canonical D-box sequences (discussed below) (figure 5*a*,*c*). Notably, Cdk1-phosphorylation at P2 (Pro is common at P3) negatively regulates APC/C-dependent substrate ubiquitination, for example Dbf4 [122], possibly due to the electrostatic repulsion between a phosphate group at P2 and Glu87. Thus, the D box is a bipartite degron comprising a coactivator-interacting N-terminal (RxxLx[D/E][Ø]) motif and a hydrophilic C-terminal-Apc10 binding segment. Coactivator and Apc10 create a D-box co-receptor for recognition of the bipartite degron. The atomic resolution structures of D-box motifs engaged by coactivators alone [69,71] and in complex with APC/C-coactivator complexes [60,80] rationalize the residue preferences at all 10 positions of the D box. Moreover, the preferences for an acidic residue at P6 and basic residue at P2 are consistent with the promotion of substrate ubiquitination by D-box phosphorylation at P6 [134] and substrate stabilization by phosphorylation at P2 [122].

*KEN box.* Another APC/C degron, the KEN motif ([DNE]KENxxP), is commonly present in APC/C substrates usually in addition to the D box [135]. Efficient ubiquitination by either APC/C^Cdc20^ or APC/C^Cdh1^ of substrates harbouring both D and KEN boxes is dependent on both degrons [54,64]. By forming a 3_10_ helix, the three consecutive residues of the KEN box face in the same orientation and engage the top surface of the β-propeller (figures 3*b* and 4*c*) [69–71]. The KEN box is usually immediately C-terminal to acidic residues (figure 5*b*,*d*), and the structure of the KEN box–coactivator complex suggested that these would engage a positively charged patch on the β-propeller. A frequently observed Asp or Asn residue at P-1 stabilizes the KEN box conformation by forming a hydrogen bond to the Asn of the KEN box (figure 4*c*) [71]. Proline residues one to two residues C-terminal of the KEN box would direct the polypeptide chain away from the surface of the β-propeller.

*ABBA motif.* The A motif was discovered in the *S. cerevisiae* Cdh1 inhibitor Acm1 [127,136]. Later bioinformatics studies identified the ABBA motif as a general class that includes the A motif as a six-residue motif (Fx[ILV][FY]x[DE]) common to vertebrate cyclin A (and *S. cerevisiae* Clb5), BubR1, Bub1 and Acm1 [120–122]. Although the A motif was originally thought to confer specificity for *S. cerevisiae* Cdh1 [71,127], the situation is more complicated. Cdc20 also binds the ABBA motif—variations in non-consensus residues confer the specificity for *S. cerevisiae* Cdh1. Glu65(P5) of the ABBA motif of Acm1 contacts Lys333 in *S. cerevisiae* Cdh1 that is a Thr in *S. cerevisiae* Cdc20 [121]. Residues of human Cdc20 required for ABBA motif binding are not conserved in human Cdh1 (although are conserved in *S. cerevisiae* Cdh1), explaining the inability of human Cdh1 to recognize the ABBA motif [121]. A structure of Acm1 in complex with *S. cerevisiae* Cdh1 revealed that the ABBA motif forms an extended structure and binds to the inter-blade groove between β-blades 2 and 3, through a related mechanism to the D box (figure 4*d*). The side-chains of the three conserved non-polar residues anchor the ABBA motif to the ABBA-motif binding groove, with the Asp at P6 forming a salt-bridge with an Arg of blade 2 [71].

*Non-canonical degrons.* In addition to the D box, KEN box and ABBA motif, non-canonical degrons have also been identified (figure 5*c*). However, some of these are likely to be variants of the well-characterized D box and KEN box degrons [71,120]. For example, the conserved Arg (P1) at the N-terminus of the D box can be substituted with Lys, His or Gln although this is often accompanied by a Lys at P7 which can interact with the acidic patch at the N-terminus of the D-box binding channel [71]. The O box identified as an APC/C degron in Orc1 closely matches the D-box consensus [137], suggesting it may interact with the D-box receptor [71], consistent with the ability of a D-box peptide to interfere with O-box recognition by APC/C^Cdh1^ [137]. A D-box peptide also inhibited APC/C^Cdh1^-catalysed ubiquitination of the Spo13 [138] and Cin8p [139], substrates that harbour non-canonical D-box motifs (figure 5*c*) [71]. Peptides modelled on the non-canonical D-box motifs of Cin8p, the O box and Spo13 inhibited the D-box-dependent ubiquitination of the budding yeast substrate Hsl1, consistent with the idea that these motifs interact with the D-box receptor of APC/C^Cdh1^ [71]. In mammals, the CRY box (CRYxPS) within the NTD of Cdc20 mediates APC/C^Cdh1^-dependent Cdc20 destruction in oocytes and embryos [140]. Insights into how the CRY box might interact with Cdh1 were provided by cryo-EM structures of the APC/C^MCC^ [92,93] (discussed in §8). These showed that the CRY box of the MCC Cdc20 subunit interacts with the WD40 domain of Cdc20 of APC/C^Cdc20^ in proximity to the D-box binding site.

In addition to modulation of APC/C–substrate affinities by substrate phosphorylation in or adjacent to the degron, ubiquitination of Lys residues within or in close proximity to degrons may influence APC/C–substrate affinities. One example of this is that the KEN-box Lys residue is one of the most frequently ubiquitinated sites *in vivo* [141]. Modification of the KEN box would be expected to reduce APC/C–substrate affinities.

Discovery of new APC/C substrates will be facilitated by high-throughput automated approaches based on protein micro-arrays such as the extract-based functional assays [142,143].

## 4. The APC/C pairs with two E2s to assemble polyubiquitin chains

The APC/C is a RING domain E3 ligase. RING domains interact directly with their canonical E2s and bring these into close proximity with substrates bound to degron recognition sites situated elsewhere on the E3 ligase [144]. Metazoan APC/C assembles atypical Lys11-linked chains to promote proteolysis and mitotic exit [145,146], in a process involving two distinct E2 activities. Chain formation is initiated with the E2 UbcH10 (also termed Ube2C) [147,148], whereas Ube2S is primarily responsible for chain extension [149–152]. Ube2S interacts with the acceptor ubiquitin to generate Lys11-linked chains through a substrate-assisted catalytic mechanism in which Glu34 on the acceptor ubiquitin activates and orients the target Lys11 to attack the donor ubiquitin conjugated to Ube2S [152]. UbcH10 and Ube2S act in concert to generate branched chains (mixed K11 and K48 linkages). The ubiquitin chain topology determines the efficiency of proteasome-dependent proteolysis of the ubiquitinated substrate [153–156]. UbcH10 alone is competent to generate short ubiquitin chains of mixed K11, K48 and K63 linkage [157,158]. Neither UbcH10 nor Ube2S are essential, suggesting an alternative E2 can function in place of UbcH10 *in vivo*, likely to be UbcH5 [159]. However, Ube2S is essential for optimal release from a SAC-dependent arrest, possibly due to its role in reactivating the APC/C on cessation of SAC signalling [149–151]. In *S. cerevisiae* the APC/C generates canonical Lys48-linked chains also using two E2s: the initiating E2 Ubc4 and the elongating E2 Ubc1 [160]. A UBA domain in Ubc1 is required for processivity [160] by enhancing Ubc1 association with the APC/C in competition with Ubc4 [161].

### 4.1. Monoubiquitination catalysed by UbcH10

**Figure 6.**
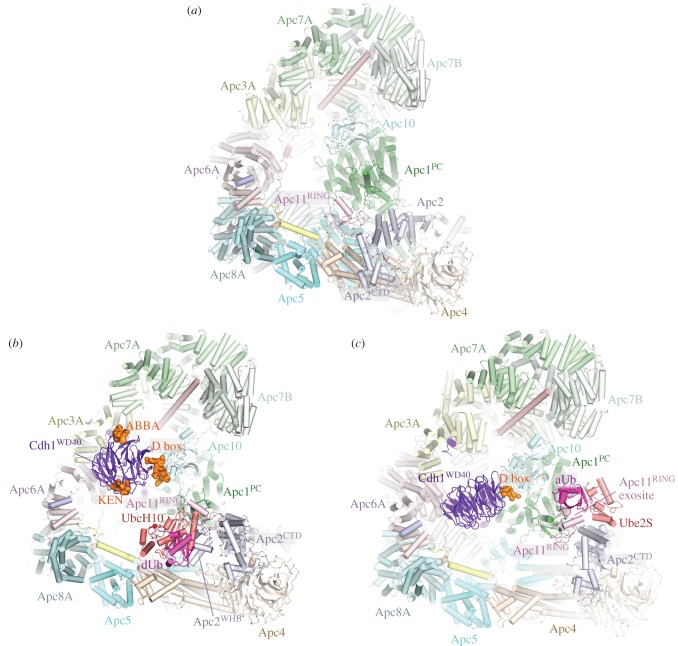
APC/C ubiquitination reaction. (*a*) Apo APC/C. In the absence of coactivator the catalytic module adopts a ‘down’ inactive conformation. UbcH10 binding to Apc11^RING^ is blocked by Apc5, and Apc5 prevents the correct location of Apc2^WHB^ required to engage UbcH10. EM density for Apc11^RING^ is weak indicating RING domain flexibility. PDB 5G05 from Zhang *et al.* [60]. (*b*) Complex of APC/C^Cdh1.substrate^ with a UbcH10 ∼ ubiquitin conjugate. Apc2^WHB^ becomes ordered and engages UbcH10. dUb: modelled donor ubiquitin conjugated to UbcH10. The C-terminus of dUb is indicated with a red sphere. PDB 5A31, from Chang *et al.* [80]. PDB for Apc2^WHB^ 4YII Chang *et al.* [111]. (*c*) APC/C^Cdh1.substrate^-Ube2S∼Ub complex. Ube2S is partially built. aUb: acceptor ubiquitin bound to the Apc11^RING^ exosite. PDB 5L9T, from Brown *et al.* [112]. The figure is based on previous work [60,80,111,112].

**Figure 7.**
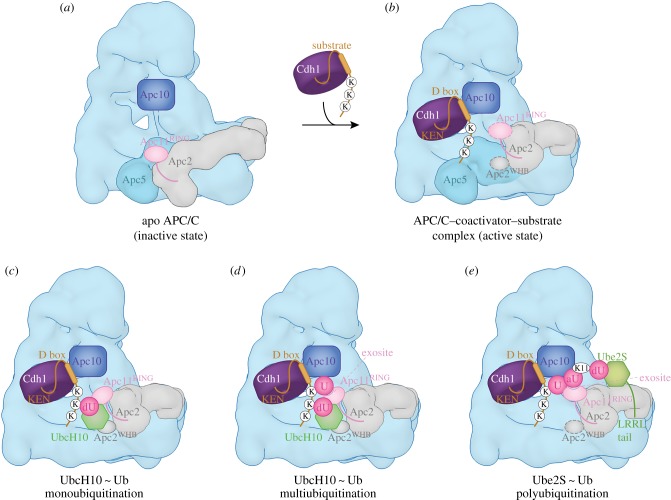
Schematic of ubiquitination reaction catalysed by the APC/C. (*a*) In the apo state, the downward position of the catalytic module would cause a clash between Apc5 and both UbcH10 and Apc2^WHB^ (as in the APC/C^Cdh1.substrate^–UbcH10 ∼ ubiquitin complex). (*b*) Binding of coactivator shifts the catalytic module (Apc2 and Apc11) to an upward position. Apc2^CTD^ together with Apc2^WHB^ and Apc11^RING^ are highly flexible. Target lysines on the APC/C substrate are shown as ‘K’. (*c*) UbcH10-catalysed monoubiquitination. dU: UbcH10-conjugated donor ubiquitin. Apc2^WHB^ rigidifies by binding to UbcH10, Apc11^RING^ is less flexible. (*d*) UbcH10-catalysed multiubiquitination. The substrate-conjugated ubiquitin (U) engages the ubiquitin-binding exosite of Apc11^RING^. (*e*) Ube2S-catalysed polyubiquitination. The distal acceptor ubiquitin (aU) of the polyubiquitin chain engages the ubiquitin-binding exosite of Apc11^RING^ positioning Lys 11 adjacent to the catalytic site of Ube2S. dU: donor ubiquitin conjugated to Ube2S. Dashed lines around Apc11^RING^ and Apc2^WHB^ denote conformational flexibility. Based on schemes from Brown *et al.* [112] and Chang & Barford [162].

Cryo-EM studies of human APC/C^Cdh1^ in complex with UbcH10 and Ube2S with and without ubiquitin have provided detailed mechanistic insights into the process of substrate ubiquitination [80,99,111,112]. UbcH10 is a canonical E2 that interacts with the RING domain of Apc11 [80,111]. In human APC/C, the catalytic module is a region of conformational flexibility [60,86]. Binding of UbcH10, but not Ube2S, is dependent on a conformation change mediated by the coactivator subunit (figures 6 and 7) [86,110]. Thus, coactivators are required for both substrate recognition and for stimulating the catalytic activity of the APC/C [117]. This conformational change involves a movement of the catalytic module from a ‘down’ to an ‘up’ position. In the ‘down’ position, Apc11^RING^ is in contact with Apc5 of the platform, blocking the UbcH10-binding site. On conversion to the coactivator-bound state, movement of the catalytic module to an upward position exposes the UbcH10-binding site on Apc11^RING^-Apc2^WHB^, resulting in at least a 10-fold increased affinity for UbcH10 [86]. In this state the catalytic module is flexible with weak density recovered and conformational heterogeneity for a variety of ternary complexes [60,86]. Coactivators also increased the catalytic efficiency of *S. cerevisiae* APC/C (decrease in *K*_m_ and increase in *V*_max_) [163], although this may result from a mechanism other than a coactivator-induced conformational change (D Barford & E Vázquez Fernández 2017, unpublished data).

The interaction of the zinc binding region (ZBR) domain of the inhibitor Emi1 with Apc11^RING^ stabilizes the conformation of the catalytic module because the ZBR domain bridges Apc1^PC^ with Apc11^RING^ and Apc2^CTD^ (figure 1*a*). This allowed definition of Apc11^RING^ and Apc2^CTD^ to a local resolution of approximately 6 Å [80] and it showed for the first time how Apc11^RING^ interacts with Apc2^CTD^. The juxtaposition of Apc11^RING^ and Apc2^CTD^ is similar to the swung out conformation of Rbx1^RING^ in activated Cul5-Rbx1 [164]. Engagement of UbcH10 with Apc11^RING^ is essentially similar to other RING domain–E2 interactions (figure 6*b*) [80,111]. Density for UbcH10 was poorly resolved, probably due to the low stoichiometry of UbcH10–APC/C interactions and conformational flexibility of the catalytic module. The Apc11^RING^–UbcH10 interface was confirmed by a detailed mutagenesis study by Schulman and colleagues [111]. On interacting with UbcH10, the catalytic module rotates by 12° relative to its position in the APC/C^Cdh1.Emi1^ complex [80]. Importantly no EM density was visible for ubiquitin in the APC/C^Cdh1^-UbcH10∼ubiquitin cryo-EM maps [80,111]. This would indicate that the ubiquitin moiety must be mobile, and only transiently adopts the closed E2∼ubiquitin conformation that primes the E2∼ubiquitin thioester bond to stimulate the intrinsic catalytic activity of E2∼ubiquitin [152,165–169]. Formation of the closed E2∼ubiquitin conformation, where the ubiquitin moiety interacts with the RING domain through its Ile36 and E2 through its Ile44, as a requirement for optimal substrate ubiquitination, is based on the finding that mutating either Ile36 or Ile44 residues in ubiquitin virtually eliminated ubiquitination of APC/C substrates [80]. The APC/C is reminiscent of other single domain RING and U-box E3s that bias the E2∼ubiquitin conformation from multiple extended states to the closed state [168,170]. As discussed elsewhere [80,111], an interesting possibility is that substrate initiation motifs that promote lysine ubiquitination [158] may induce a closed UbcH10∼ubiquitin conformation.

The study of Schulman and colleagues revealed that Apc2^WHB^ forms an unusual interaction with the backside of UbcH10 [111]. This interaction follows a rigidification of the WHB domain (which is mobile in UbcH10-free structures) induced upon UbcH10 binding (figure 7*c*). Apc2^WHB^ is essential and specific for APC/C-UbcH10-dependent substrate modification, but is dispensable for UbcH5 activity. The activity of Ube2S, which does not interact with Apc2^WHB^, is also independent of Apc2^WHB^ [111]. Apc2^WHB^ both enhances APC/C-UbcH10 affinity, but importantly also greatly stimulates (by more than 100-fold) the catalytic activity of UbcH10, likely by stabilizing the E2∼ubiquitin closed conformation through an allosteric mechanism. Since the WHB-binding interface of UbcH10 differs substantially from its counterpart in UbcH5, similar interactions between UbcH5 and Apc2^WHB^ are not possible, thus explaining how Apc2^WHB^ contributes to UbcH10 specificity [111].

### 4.2. Polyubiquitination catalysed by Ube2S

The processive ubiquitination reaction catalysed by Ube2S involves modification of a constantly changing substrate that is the growing distal ubiquitin moiety of the polyubiquitin chain. Biochemical studies showed that UbcH10 and Ube2S do not compete for the same binding site on the APC/C [150,152], suggesting that Ube2S differs from canonical E2s by not interacting with the RING domain of Apc11, a notion also consistent with the observation that Ube2S catalyses formation of unattached K11-linked polyubiquitin chains [171]. APC/C–Ube2S interactions are dependent on the C-terminal LRRL motif of Ube2S [86,154,172,173]. The APC/C dramatically improves the catalytic efficiency of Ube2S-mediated Lys11-linked chain assembly [99,173]. This stimulatory effect of the APC/C requires a surface centred on Ala46 of the acceptor ubiquitin, indicating that APC/C tracks the distal ubiquitin of a growing ubiquitin chain [173]. This finding explains how the APC/C generates ubiquitin chains without altering its interactions with substrates and E2s.

Brown and colleagues [99] in agreement with Kelly *et al*. [173] showed that the APC/C increased Ube2S catalytic efficiency to massively increase polyubiquitination. Although this catalytic enhancement requires Apc11^RING^, two lines of evidence suggested that this did not involve the canonical E2-binding surface on Apc11^RING^. Mutagenesis studies identified a novel surface on Apc11^RING^ (termed the exosite) required for Ube2S activity, a result complemented by NMR data showing chemical shift perturbations in this region of Apc11^RING^ in the presence of ubiquitin. Conversely, acceptor ubiquitin mutants with specific defects in APC/C-Ube2S-dependent ubiquitination [99,152,173] map to a RING-binding surface on ubiquitin identified by NMR [99]. In a subsequent study, the structural basis for Ube2S-catalysed ubiquitin chain extension was defined [112]. A cryo-EM reconstruction of APC/C^Cdh1^ in complex with Ube2S revealed that the Ube2S UBC (ubiquitin conjugating) domain interacts with Apc2, rationalizing the deleterious effects of mutations of the αC and αD helices (figure 6*c*) [99,112,173]. Its LRRL C-terminus interacts at a site between Apc2 and Apc4, as previously determined for the Emi1 LRRL tail in the APC/C^Cdh1.Emi1^ structure [80]. The distal (acceptor) ubiquitin moiety of the ubiquitinated substrate engages the repurposed exosite on Apc11^RING^, following a conformational change of Apc11^RING^, presenting its K11 residue to undergo nucleophilic attack onto the donor ubiquitin conjugated to Ube2S. Thus the Apc11^RING^ exosite captures the tip of the growing polyubiquitin chain promoting its reaction with Ube2S∼ubiquitin bound to Apc2 (figure 7*e*).

The relative locations of the UbcH10 and Ube2S binding sites on the APC/C also fit with their different functions—priming and elongation, respectively (figures 6 and 7). UbcH10 is located closer to the degron binding site on the substrate-recognition module, facing into the central cavity, and this is consistent with the relatively close proximity of the preferred target lysines to APC/C degrons (figures 6*b* and 7*c*). In contrast, Ube2S is sited on the periphery of the molecule, able to accept the distal ubiquitin moiety on the polyubiquitin chain. The growing polyubiquitin chain can then be easily accommodated on the outside of the molecule (figures 6*c* and 7*e*).

### 4.3. Multiubiquitination catalysed by UbcH10

The repurposing of Apc11^RING^ that stimulates Ube2S-catalysed ubiquitin chain extension also plays a role in protein multi-ubiquitination catalysed by UbcH10 through its interaction with the canonical E2-binding site on Apc11^RING^. A cryo-EM structure of a monoubiquitinated substrate bound to APC/C^Cdh1^-UbcH10∼ubiquitin showed that the substrate-conjugated ubiquitin moiety interacted with the Apc11^RING^ exosite [112], a finding supported by mutagenesis data revealing that multi-ubiquitination catalysed by UbcH10 was defective in the Apc11^RING^ exosite mutant. The structure suggests a model for how an interaction between the Apc11 exosite and a substrate conjugated ubiquitin would increase substrate affinity and hence processivity (figure 7*d*). Importantly, these data are consistent with the proposal that substrate ubiquitination primes APC/C substrates for further ubiquitination through a mechanism termed processive affinity amplification [174].

The inherent weak affinities between the APC/C–substrate complex and the E2s UbcH10 and Ube2S were overcome by employing artificial reinforcement of these interactions through a three-way chemical linkage involving the substrate, ubiquitin and E2 [111,112]. The interactions between the Apc11^RING^ exosite and ubiquitin were strengthened by generating a ubiquitin variant (Ubv) with substantially increased affinity for Apc11^RING^ [112]. In another approach to stabilize APC/C^Cdh1.substrate^ interactions with UbcH10, either UbcH10 was directly fused to the C-terminus of Apc11 or the LRRL tail of Ube2S was fused to the C-terminus of UbcH10, enhancing its affinity 10-fold [80].

## 5. The APC/C controls cell-cycle-dependent substrate degradation

The capacity of the APC/C to control the degradation of regulatory proteins in a cell-cycle-dependent manner defines the ordered progression through distinct phases of the cell cycle. The factors that affect differential rates of protein degradation during the cell cycle depend upon both changes in the composition and conformation of the APC/C itself as well as direct changes to individual substrates, and their intrinsic processivity. Switching between Cdc20 and Cdh1 contributes to altering APC/C substrate specificity. Cdh1 directs APC/C-mediated ubiquitination of the Aurora kinases [118], which are not substrates of APC/C^Cdc20^. Nevertheless, apart from this example, there are relatively few instances known where the timing of substrate degradation can be directly explained by the switch of coactivator. Apart from coactivator switching, the two best-characterized regulatory mechanisms for determining the cell cycle order of APC/C-regulated substrate degradation are the spindle assembly checkpoint and substrate phosphorylation.

### 5.1. Substrate degradation at the spindle assembly checkpoint

A few APC/C substrates are degraded in early mitosis (prometaphase), for example Nek2A, cyclin A and Hox10, during an active SAC [175–181]. Thus, ubiquitination of these substrates is not inhibited by the SAC. These substrates differ from the canonical D-box and KEN-box-dependent substrates cyclin B and securin whose ubiquitination is inhibited by the MCC [24,177,178]. This implies that these early substrates would incorporate additional novel APC/C-recognition motifs that do not rely on binding to D-box and KEN-box receptors. Indeed, in the case of Nek2A, its interaction with the APC/C occurs in the absence of coactivators [182,183], through a C-terminal Met-Arg (MR) tail motif that mimics the IR tail of coactivator and Apc10 [182,183]. However, coactivators are required to mediate Nek2A ubiquitination [117,181] by inducing a UbcH10-binding site on the APC/C [86]. For Nek2A to be degraded during an active checkpoint it requires both its C-terminal MR tail and the adjacent leucine zipper, implying a requirement for Nek2A dimerization. Deletion of either motif shifts the degradation to anaphase that is KEN-box dependent [181,184]. Nek2A binds to apo APC/C, but not APC/C^MCC^ [181], and its binding required the C-box site of Apc8, likely through its MR tail (since the IR tail of Cdc20 of the MCC binds to the C-box binding site of Apc8A [92,93]).

Cyclin A is degraded soon after nuclear envelope breakdown (NEBD) in prometaphase some 20 min before cyclin B. Importantly cyclin A degradation is not inhibited by an active SAC, although its degradation is affected by the SAC [121,176–178]. When the SAC is repressed by the over-expression of a dominant negative BubR1 mutant, cyclin B1 is degraded shortly after NEBD, similar to cyclin A [177]. In further support that the SAC is a major cause of the difference in timing of cyclin A and cyclin B degradation, inactivating the SAC using the Mps1 kinase inhibitor reversine caused premature cyclin B degradation, with kinetics similar to cyclin A, and importantly no longer dependent on Apc15 [59], which is required to reactivate APC/C^Cdc20^ when the SAC is switched off.

Both the N-terminal 165 residues of cyclin A and the Cks subunit are necessary and sufficient to confer the SAC-resistant degradation of cyclin A [178,185,186]. Deletion of the cyclin A D box does not stabilize the protein at prometaphase, or affect degradation timing later in mitosis, questioning the importance of this motif in APC/C-dependent recognition [177,178,182,187]. A region of cyclin A (residues 98–165) C-terminal to the D box contributes to the degradation timing and this region (which incorporates the ABBA motif [121]) binds directly to Cdc20, competing with BubR1 [186]. An ABBA motif also contributes to the early timing of Clb5 degradation in *S. cerevisiae* compared with securin and Dbf4 [122]. However, unlike vertebrate cyclin A2, Clb5 degradation is sensitive to the SAC although there exists a low rate of Clb5 degradation during a SAC that depends on the ABBA motif [122].

The ABBA motif clearly plays a role in determining the early destruction of cyclin A2 and Clb5 relative to cyclin B and securin. However, this may not be entirely due to the ability of the ABBA motif to overcome the SAC-induced inhibition of D-box and KEN-box-dependent substrates. One possibility is that cyclin A2 is a more processive substrate. This could be explained if cyclin A2 has a relatively higher affinity for the APC/C, thus competing effectively for binding sites on the APC/C. The ABBA motif may contribute to the higher affinity. However, against the competition argument is the finding that in *S. cerevisiae* over-expression of Clb5 did not alter the relative timing of destruction of the later substrate securin [188]. It is also interesting that in inactivated *Xenopus* egg extracts (where there is a weak checkpoint), mutation of the ABBA motif (**F**x[I/L/V][F/Y]x**VD**: residues mutated in bold) to Ala had no to little effect on cyclin A degradation [189].

The Cks subunit of the Cdk1–cyclin B1–Cks complex recruits the complex to the checkpoint-inhibited phosphorylated APC/C at prometaphase, but ubiquitination of cyclin B1 is blocked by the MCC. This prior binding renders cyclin B1 a better APC/C substrate in metaphase [25].

### 5.2. Phosphorylation can regulate the timing of substrate ubiquitination

Phosphorylation of D box and KEN box degrons has important consequences for controlling the timing of APC/C-mediated protein degradation. Cdk1-dependent phosphorylation of the P2 site of Dbf4 suppresses its destruction [122], contributing to the timing of its destruction in mitosis. A bulky negatively-charged residue at P2 interferes with D-box binding to the D-box receptor of the coactivator whereas phosphorylation at the P6 position promotes human securin degradation [134]. The structural explanation for this was discussed in §3. In contrast, Cdk1-mediated phosphorylation of *S. cerevisiae* securin in close proximity to the KEN box (17 residues C-terminal) and D box (14 residues N-terminal) reduces the rate of APC/C-dependent securin ubiquitination some 5–10 fold [190]. Dephosphorylation of these sites by Cdc14 therefore promotes securin degradation. Interestingly, since active separase (produced as a result of securin degradation) stimulates Cdc14, a positive feedback loop is generated involving Cdc14-mediated dephosphorylation of securin. Together with the partial inactivation of Cdks at metaphase due to APC/C^Cdc20^-mediated destruction of mitotic cyclins, it increases the rate of securin degradation and the abruptness of anaphase onset [122,190]. In *S. cerevisiae*, one factor delaying securin degradation relative to Clb5, even in the absence of the SAC, is Cdk1-dependent phosphorylation of residues proximal to its KEN box.

At S-phase, Cdk-dependent phosphorylation of amino acids in the immediate vicinity of the D box of Cdc6 blocks binding to the APC/C, thereby protecting Cdc6 from ubiquitination, and promoting DNA replication origin licensing [191]. In another example, Aurora A-kinase phosphorylation of the D-box P3 residue stabilizes geminin [192], likely because the P3 position has a preference for non-bulky residues.

### 5.3. Substrate ubiquitination topology may affect timing of proteolysis

The pattern of substrate ubiquitination (multi, poly and branched chains) that favours proteasome-dependent proteolysis (and possibly inhibition of DUB activity) would also contribute to more effective substrate destruction [153,154]. Processively polyubiquitinated substrates are degraded earlier in the cell cycle [122,155,193,194]. It is possible that the position of degrons relative to target lysines affects the efficiency and type of protein ubiquitination.

Finally, in mitosis, the mitotic spindle regulates the timing of spindle assembly factor (SAF) degradation through the microtubule-mediated protection of SAF ubiquitination [195].

## 6. Phosphorylation regulates APC/C activity at multiple levels

### 6.1. APC/C phosphorylation promotes Cdc20 association and activation

APC/C activity is entirely dependent on its association with either of the two coactivators Cdc20 and Cdh1, with the APC/C being activated early in mitosis (after NEBD—prometaphase), remaining active until late G1. Although high mitotic Cdk activity is required to stimulate the APC/C in mitosis, the APC/C remains active after mitotic cyclin degradation. This is due to the reciprocal effects of Cdk phosphorylation on the activities of Cdc20 and Cdh1 through affecting their affinity for the APC/C. The association of Cdc20 and Cdh1 with the APC/C is controlled at the level of both the core APC/C and coactivator phosphorylation. Cdk-dependent phosphorylation of core APC/C subunits activates APC/C^Cdc20^ [196–201] by promoting Cdc20 association [60,199,201–203], whereas Cdh1 binding does not require APC/C phosphorylation [60,198]. Simultaneously, Cdk phosphorylation of Cdh1 completely blocks its capacity to bind and activate both mitotic and interphase APC/C [32,80,198,204]. As Cdk activity declines at anaphase due to APC/C^Cdc20^-mediated ubiquitination of cyclin A and cyclin B, both the APC/C and Cdh1 become dephosphorylated. This inactivates Cdc20, but allows binding of Cdh1 to generate APC/C^Cdh1^. Cdh1 is inactivated in late G1 due to S-phase cyclin-dependent phosphorylation and Emi1.

Multiple APC/C subunits are phosphorylated in early mitosis associated with activation of APC/C^Cdc20^. Apc1 and Apc3 are hyper-phosphorylated, with Apc3 phosphorylation readily detected by its retarded mobility on SDS-PAGE. Phosphorylation mapping by mass spectrometry of endogenous APC/C defined multiple phosphosites on Apc1 and Apc3 [201,202,205–207], findings confirmed by *in vitro* APC/C phosphorylation analysis using purified Cdk and Plk1 [60]. Two hyper-phosphorylated regions of Apc1 and Apc3 are the 300s loop of the Apc1 WD40 domain (Apc1^300s^ loop), and a 300-residue segment in Apc3.

In 2016 three studies provided insights into mechanisms of activation of vertebrate (human and *Xenopus*) APC/C^Cdc20^ by mitotic phosphorylation. These studies revealed that phosphorylation-dependent APC/C^Cdc20^ activation primarily involves phosphorylation of the Apc1^300s^ loop that relieves an auto-inhibitory segment within the Apc1^300s^ loop, thereby enabling Cdc20 association [60,202,203]. Introducing phosphomimetics into this loop stimulated the ability of Cdc20 to activate the APC/C [60,202,203] and promoted Cdc20 binding [202] in the absence of APC/C phosphorylation. In contrast, mutating phosphosites to Ala ablated Cdc20-dependent APC/C activation [202,203] and Cdc20 binding [203].

**Figure 8.**
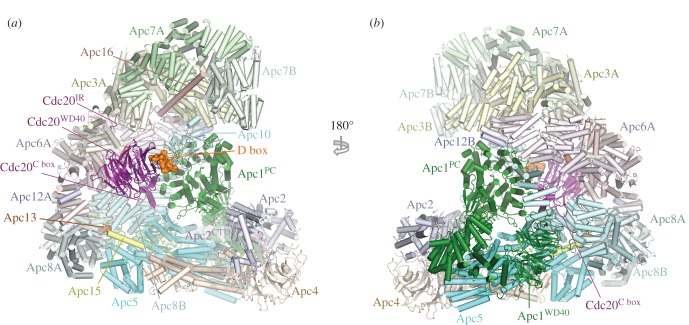
Overall structure of the phosphorylated APC/C^Cdc20.substrate^ complex. (*a*) and (*b*) Two orthogonal views of the APC/C^Cdc20.substrate^. The substrate is the high affinity budding yeast substrate Hsl1 (residues 667 to 872 containing a D box and KEN box). EM density for Apc11^RING^ is weak indicating RING domain flexibility. PDB 5G04, from Zhang *et al.* [60].

**Figure 9.**
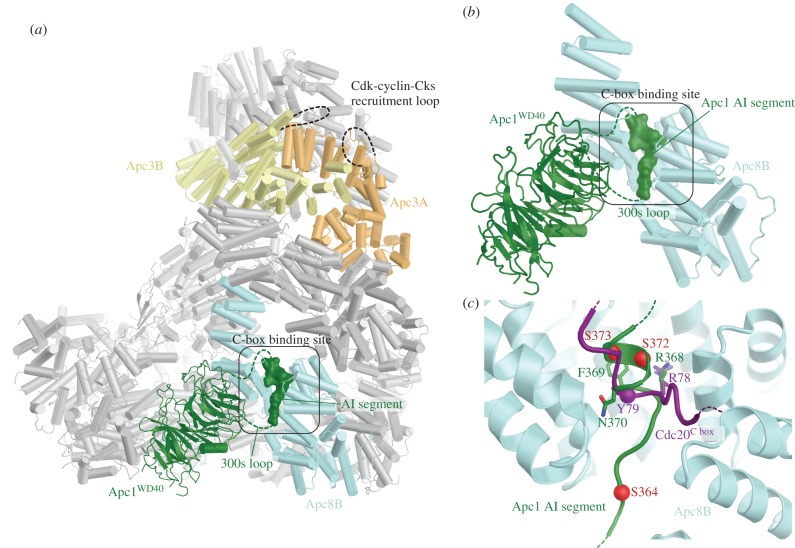
Control of APC/C^Cdc20^ by phosphorylation. (*a*) In the unphosphorylated state an auto-inhibitory segment (AI; dark green) within the Apc1^300s^ loop of Apc1^WD40^ mimics the Cdc20 C-box motif and binds to the C-box binding site, blocking Cdc20 association. The AI segment is located on the same face of the APC/C as the hyperphosphorylated Apc3 loop. (*b*) Zoomed view of the AI segment of Apc1^WD40^ associated with the C-box binding site of Apc8B. (*c*) Superposition of the AI segment with the Cdc20 C box (purple) shows that a conserved Arg residue anchors both the C box and the AI segment to the C-box binding site. Sites of mitotic phosphorylation present within the AI segment that activate APC/C^Cdc20^ are depicted as red spheres. From Zhang *et al.* [60].

To understand the molecular basis for how phosphorylation activates APC/C^Cdc20^, a cryo-EM structure of phosphorylated APC/C^Cdc20^ was determined [60]. The structure of phosphorylated APC/C^Cdc20^ is very similar to that of unphosphorylated APC/C^Cdh1^ (figures 1 and 8). Cdc20 interacts with the APC/C through three motifs: the C box to Apc8B (augmented by the KILR motif [208]), the IR tail to Apc3A and a region contacting Apc1^PC^. Relative to Cdh1 the contacts are fewer. Strikingly, EM density corresponding to phosphorylated regions could not be observed, indicating that phosphorylated regions of the APC/C do not directly or indirectly contribute to increasing the affinity of the APC/C for Cdc20. This implied that APC/C phosphorylation would remove an inhibitory segment from a Cdc20 binding site. To explore this possibility, the structures of phosphorylated and unphosphorylated apo APC/C were compared. The two structures were very similar, except that in the unphosphorylated apo structure, a segment of EM density occupies the C-box binding site (figure 9). The proximity of this unassigned EM density to the disordered 300s loop of the Apc1 WD40 domain (Apc1^WD40^) suggested that this segment corresponded to a region of the Apc1^300s^ loop. In a structure determined with this loop deleted, the C-box binding site was devoid of EM density [60]. Deletion of the Apc1^300s^ loop constitutively activated APC/C^Cdc20^ and phosphorylation did not further enhance activity [60], a finding made independently by Kraft *et al.* [202]. These data convincingly showed that a region within the Apc1^300s^ loop (an auto-inhibitory (AI) segment) represses Cdc20 stimulation of unphosphorylated APC/C activity, further supported by data in Li *et al.* [110]. Phosphorylation releases this auto-inhibition. In support of the idea that direct phosphorylation of the AI segment releases this auto-inhibition, substituting Glu for Cdk phosphorylation sites within the AI segment constitutively activated APC/C^Cdc20^ [60].

**Figure 10.**
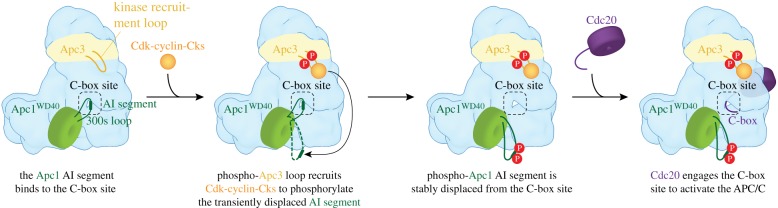
Schematic of control by phosphorylation. In the unphosphorylated state an auto-inhibitory (AI) segment of Apc1^WD40^ mimics the Cdc20 C-box motif and binds to the C-box binding site, blocking Cdc20 association. Initial Cdk-dependent phosphorylation of a kinase recruitment loop in Apc3 recruits Cdk-cyclin-Cks to the APC/C to facilitate intramolecular phosphorylation of the AI segment (when transiently displaced from the C-box binding site). The phosphorylated AI segment is stably displaced from the C-box binding site, permitting Cdc20 association to generate APC/C^Cdc20^. This scheme indicates the relay mechanism by which initial phosphorylation of exposed consensus Cdk1 sites on Apc3 allow recruitment of Cks-Cdk-cyclin to the APC/C to promote intramolecular phosphorylation of Apc1. From Zhang *et al.* [60].

The AI segment includes an Arg-Phe dipeptide, analogous to the Arg-Tyr motif of the C box. Modelling of the AI segment into EM density showed that the Arg side chain of the AI segment mimics the Arg of the Arg-Tyr motif of the C box, anchoring the AI segment to the C-box binding site (figure 9*c*). Mitotic phosphorylation of sites flanking the Arg-Phe motif would destabilize interactions between the AI segment and the C-box binding site through steric hindrance and charge repulsion, leading to the displacement and disordering of the AI segment and relief of auto-inhibition. These findings that an auto-inhibitory segment within the Apc1^300s^ loop blocks Cdc20 activation and that its mitotic phosphorylation relieves this auto-inhibition are in agreement with biochemical data [202,203] (figure 10). Fujimitsu and colleagues [203] showed that Apc1^300s^ bound to the APC/C in an anaphase extract, whereas the phosphomimetic mutants abolished this interaction, highlighting how the interaction of Apc1^300s^ with the APC/C is dependent on its phosphorylation status.

The data of Zhang *et al.* [60] indicated that the critical determinant of activation of APC/C^Cdc20^ by mitotic phosphorylation was displacement of the AI segment to relieve auto-inhibition. However, Apc3 is also highly phosphorylated in mitosis [201,202,205–207] and Cks stimulates both Cdk-dependent activation of APC/C^Cdc20^ [197,209] and Apc1 and Apc3 phosphorylation [60,209], and interacts with Apc3 [25,203,209,210]. Deletion of the hyperphosphorylated Apc3 loop reduced both Apc1 AI segment phosphorylation [60] and APC/C^Cdc20^ activation [60,203], as well as disrupting interactions between the APC/C and Cdk–cyclin A–Cks [60,203]. The phosphorylated Apc3 loop (residues 202–342) directly binds Cks [203]. Thus a likely explanation for these findings, and for the lag phase that accompanies APC/C activation by Cdk1–cyclin B–Cks [197], is that Apc3 phosphorylation recruits Cdk–cyclin–Cks through Cks [25,209,210] to stimulate Apc1 auto-inhibitory segment phosphorylation via a relay mechanism. Cdk–cyclin–Cks association with the Apc3 loop would allow for a kinetically more efficient intra-molecular phosphorylation of the Apc1 auto-inhibitory segment that only becomes accessible to Cdk when transiently displaced from the C-box binding site. Phosphorylation of the Apc1^300s^ loop stably displaces the AI segment from the C-box binding site (figure 10). Intra-molecular phosphorylation of the Apc1^300s^ loop is associated with relaxed Cdk specificity and the phosphorylation of non-consensus Cdk sites [60]. Interestingly these sites are not evolutionarily conserved, suggesting that the exact location of the phosphorylation sites with Apc1^300s^ is not critical to their capacity to displace the AI segment.

Cdh1 and Cdc20 bind to common sites on the APC/C, yet only APC/C^Cdc20^ is activated by phosphorylation [80,60]. The phosphorylation-independent activity of Cdh1 is due to the increased affinity of Cdh1 for unphosphorylated apo APC/C, which overcomes the inhibition from the unphosphorylated AI segment. The increased affinity results from the more extensive contacts formed between the APC/C and Cdh1 relative to Cdc20. This also explains why the APC/C inhibitor TAME [211], which interacts with both the IR tail and C-box binding sites through structural mimicry of the IR tail and C box, is a more potent inhibitor of APC/C^Cdc20^ than APC/C^Cdh1^ [60].

### 6.2. Cdk phosphorylation of Cdh1 and Cdc20 inhibits APC/C association

Binding of Cdh1 to the APC/C is negatively regulated by phosphorylation. Based on the structure of APC/C^Cdh1.Emi1^, the four phosphorylation sites (Ser40, Thr121, Ser151 and Ser163 of human Cdh1) that suppress Cdh1 activity [80,212] can be rationalized (figure 3*c*). Phosphorylation of individual sites only partially suppresses APC/C activity, whereas phosphorylation of all four sites would destabilize Cdh1^NTD^–APC/C interactions through electrostatic repulsion and steric clashes. Ser40 is immediately N-terminal to the C box, whereas the side-chains of Ser151 and Ser163 flank the KLLR motif [80].

Cdk phosphorylation of the Cdc20 NTD also negatively regulates Cdc20 activation and its binding to the APC/C [115,213,214]. Cdk2-cyclin A2 phosphorylation of Cdc20 at interphase is proposed to prevent premature activation of APC/C^Cdc20^, thereby stabilizing cyclin B1 and promoting mitotic entry [214]. In mitosis Cdk1-cyclin B1 may contribute to Cdc20 phosphorylation [214]. The Cdk phosphosites are close to the N-terminus of the C box (Thr55, Thr59 and Thr70 in human Cdc20, with C box comprising residues Asp77 to Arg83) [60], thus phosphorylation may block C-box binding to the Apc8B C-box binding site, reminiscent of Cdh1 inhibition by Cdk phosphorylation [80]. However, it should be noted that in the APC/C^Cdc20^ structure residues N-terminal to Ser72 are largely disordered [60], making it unclear mechanistically how phosphorylation of Cdc20 N-terminal to the C box inhibits its activity. Significantly, mutation of Thr55, Thr59 and Thr70 to Ala produced no cellular phenotype [214], suggesting that multiple Cdk phosphosites on Cdc20 contribute to its inactivation. These may involve mechanisms in addition to directly inhibiting its association with the APC/C. For instance, it is possible that Cdc20 NTD phosphorylation affects the structure of free Cdc20, possibly promoting a closed conformation that cannot bind the APC/C [215]. PP2A has been suggested as the Cdc20 phosphatase [115,214] and possibly binds directly to the APC/C mediated by PP2A^B56^ [216]. A recent study in *Caenorhabditis elegans* showed that kinetochore-associated PP1 also contributes to dephosphorylation of Cdc20 Cdk phosphosites (with Thr32, equivalent to human Thr70, being a key site responsible for the control of *C. elegans* Cdc20 by phosphorylation) through a mechanism by which Cdc20 is recruited to kinetochore by the ABBA motif of Bub1 [217]. Thus, depending on the status of their microtubule attachment, kinetochores either inactivate (via the SAC) or activate (via Cdc20 dephosphorylation) APC/C^Cdc20^. This explains the paradox that the checkpoint proteins Bub1/Bub3 promote anaphase onset independently of the checkpoint [218,219]. While Ala substitution of Cdk sites within the NTD of *C. elegans* Cdc20 accelerated normal mitosis, this mutant retained the ability to significantly delay mitosis in the presence of unattached kinetochores [217], indicating that Cdk phosphorylation of Cdc20 does not contribute to the SAC.

Plk1 (mediated through a scaffolding role of Bub1) phosphorylation of human Cdc20 on Ser92 impaired the assembly of polyubiquitin chains *in vitro*, mainly through inhibition of Ube2S [220] by preventing the association of Ube2S to the APC/C [220,221]. Analysis of the APC/C^Cdc20^ structure indicates that Ser92 is in contact with Apc8B [60], remote from the Ube2S binding site [112]. Thus the molecular mechanism by which Ser92 phosphorylation inhibits Ube2S is not currently clear. Ser92 phosphorylation does not affect the MCC-mediated inhibition of APC/C^Cdc20^, revealing that Bub1-Plk1 directly inhibits APC/C^Cdc20^ through a mechanism that is independent of the MCC [220]. The inhibitory phosphorylation on Cdc20 is removed by PP2A^B56^, a kinetochore-bound phosphatase [220,221] and PP1 [217].

### 6.3. Substrate phosphorylation can regulate association with the APC/C

Direct phosphorylation of substrates provides a third level of APC/C control by protein phosphorylation, discussed above.

## 7. Emi1 inhibits APC/C^Cdh1^

In vertebrates, Emi1 functions as an antagonist of APC/C^Cdh1^ during G2 [42,43]. Four functional elements of Emi1 mediate APC/C^Cdh1^ inhibition [80,107,172,222]. Similar to the MCC, Emi1 blocks D-box recognition by APC/C–coactivator complexes and also antagonizes the two E2s UbcH10 and Ube2S. A D-box motif that occludes substrate recognition is connected through a linker to a zinc-binding region (ZBR) (Emi1^ZBR^) that interferes with UbcH10-dependent APC/C activity [107,172,222] (figure 1*a*). A C-terminal LRRL sequence (LR tail: Emi1^LR^), identical to the LRRL motif required for Ube2S-dependent synthesis of polyubiquitin chains on APC/C substrates [150,151] and its association with the APC/C [86,154,172], antagonizes Ube2S [107,172] by interacting with the Ube2S LRRL-tail binding site on Apc4 [80].

## 8. Reciprocal regulation of the spindle assembly checkpoint and APC/C^Cdc20^

To ensure the fidelity of the inheritance of genetic information, the cell has evolved cell cycle checkpoints that control progression through cell cycle transitions that are dependent on the successful completion of a preceding event. The spindle assembly checkpoint (SAC), also known as the mitotic checkpoint and kinetochore checkpoint, coordinates sister chromatid segregation at the metaphase to anaphase transition with the correct bipolar attachment of sister chromatids to the mitotic spindle [40,41]. The SAC is exerted by the mitotic checkpoint complex (MCC), a multi-protein complex that functions to repress APC/C activity. Generation of the MCC is catalysed by unattached kinetochores whose structural and biochemical properties are becoming well defined [223,224]. MCC assembly occurs on the outer regions of the kinetochore, specifically the KMN (Knl1-Mis12-Ndc80) network which functions as a recruiting site for multiple checkpoint components. Key among these are the Mad and Bub proteins, identified over 25 years ago in genetic screens for SAC components [225,226]. A checkpoint cascade results in the assembly of a molecular scaffold that catalyses conversion of O-Mad2 (open state of Mad2) to C-Mad2 (closed state of Mad2), in a process that requires the kinetochore-associated C-Mad2. In the template-assisted mechanism [227], the kinetochore-associated C-Mad2, bound to the kinetochore through Mad1, interacts with O-Mad2 to promote its conversion to C-Mad2, a reaction catalysed by Mps1 [224,228]. C-Mad2 captures the N-terminus of Cdc20 and the resultant C-Mad2-Cdc20 binary complex interacts rapidly with BubR1-Bub3 to generate the tetrameric MCC (C-Mad2-Cdc20-BubR1-Bub3) [229]. The MCC is a potent APC/C inhibitor, some 3000-fold more potent than Mad2 alone [229]. The target of the MCC is APC/C^Cdc20^ [230].

The structural mechanisms underlying how the APC/C and the MCC are reciprocally regulated in the context of the SAC were defined from cryo-EM reconstructions of APC/C^Cdc20^ in complex with the MCC (APC/C^MCC^) [92,93]. These studies explained how the MCC blocks D-box- and KEN-box-dependent substrates from interacting with APC/C^Cdc20^, and also surprisingly revealed how the MCC interferes with the initiating E2, UbcH10.

*Overall structure of the APC/C^MCC^.* Recombinant reconstituted APC/C^MCC^ comprises two Cdc20 subunits, consistent with the notion that the MCC interacts with APC/C^Cdc20^ [92,93,230–232]. Importantly, the overall structure is essentially identical to the endogenous APC/C^MCC^ isolated from checkpoint-arrested HeLa cells (at much lower resolution) [105]. This validated the notion that the physiologically relevant form of APC/C^MCC^ includes two Cdc20 molecules (termed Cdc20^APC/C^ and Cdc20^MCC^ for the Cdc20 subunits of APC/C^Cdc20^ and MCC, respectively) [230,231]. In the APC/C^MCC^ reconstruction, a large density element termed the MCC-Cdc20 module (MCC and Cdc20^APC/C^) occupies the central APC/C cavity extending from the front side of the platform domain. The core MCC elements comprising Cdc20^MCC^, the TPR domain of BubR1 and C-Mad2 resemble their counterparts in the free *S. pombe* MCC structure [69]. Although present in the reconstituted complex, no EM density was visible for BubR1's C-terminal regions (that includes a pseudo-kinase domain) and its associated Bub3 subunit. Mad2 adopts the closed conformation with its safety belt entrapping the N-terminal KILR motif of Cdc20.

**Figure 11.**
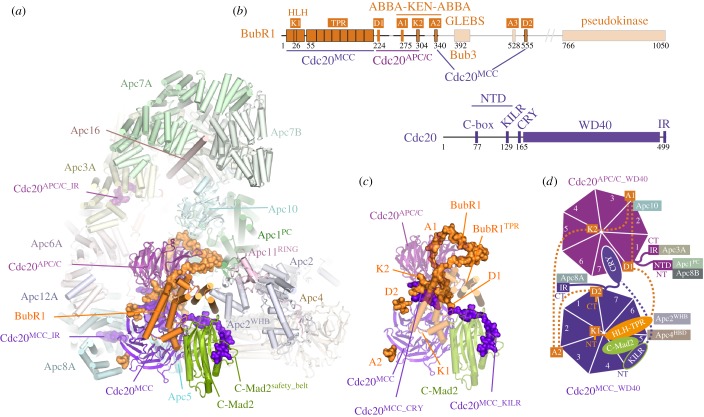
APC/C regulation by the MCC. (*a*) Atomic structure of APC/C^MCC^. Ordered regions of BubR1 C-terminal to the TPR domain are shown in space-filling representation, as is the NTD and IR tail of Cdc20^MCC^. (*b*) Schematic of BubR1 showing positions of D-box (D1, D2), KEN-box (K1, K2) and ABBA motifs (A1–A3) and schematic of Cdc20. (*c*) Details of the MCC-Cdc20^APC/C^ module with BubR1 forming extensive interactions with Cdc20^MCC^ and Cdc20^APC/C^. (*d*) Schematic of interactions formed by Cdc20^MCC^ and Cdc20^APC/C^ with BubR1 and APC/C subunits. PDB 5LCW, from Alfieri *et al.* [92].

The MCC docks into the central cavity of the APC/C contacting Cdc20^APC/C^ and Apc2^WHB^ (figure 11). Apc2^WHB^ rigidifies and repositions (relative to APC/C^Cdh1^-UbcH10 [111]) to engage BubR1. Contacts between the two Cdc20 molecules are mainly mediated by BubR1 that intertwines between them. Through extensive contacts between BubR1 and the two Cdc20 molecules, BubR1 obstructs degron dependent binding to both coactivator subunits. This is achieved because BubR1 incorporates two copies of both the D-box (D1, D2) and KEN-box motifs (K1, K2) and three copies of the ABBA motif (A1–A3) (figure 11*b*) [92,93,120,121,233]. Six of these motifs interact with the six degron recognition sites on both coactivators thereby blocking substrate recognition. Apart from the N-terminal KEN motif (K1) that is present within a structured region N-terminal to the TPR domain (within a helix-turn-helix motif) five of the pseudo-substrate degron motifs are present in a long disordered segment, C-terminal to the TPR domain (figure 11*b*). This allows BubR1 to intertwine around Cdc20^APC/C^ and then fold back to contact the A2 and D2 sites on Cdc20^MCC^, forming a lariat-like structure (figure 11*a*,*c*,*d*). The contacts between D1, A1 and K2 of BubR1 and Cdc20^APC/C^ explain why these three motifs are critical to APC/C–MCC interactions and function to sustain the checkpoint response [121,208,233–236]. In contrast, the contacts between A2 and D2 with Cdc20^MCC^ are not as critical for MCC stability and APC/C–MCC interactions, hence the more modest effects of disrupting the checkpoint when these motifs are deleted. D1, A1, K2, A2 occur in an evolutionarily conserved cassette, suggesting the mechanism for inhibiting the APC/C is conserved in all major eukaryotic super groups over one billion years of evolution [233,236]. In addition to directly blocking degron recognition sites on the Cdc20^APC/C^ WD40 domain, MCC interactions with APC/C^Cdc20^ cause a rotation and translation of Cdc20^APC/C^ away from Apc10, disrupting the D-box co-receptor, with a portion of A1 now contacting the D-box binding surface of Apc10 [92].

**Figure 12.**
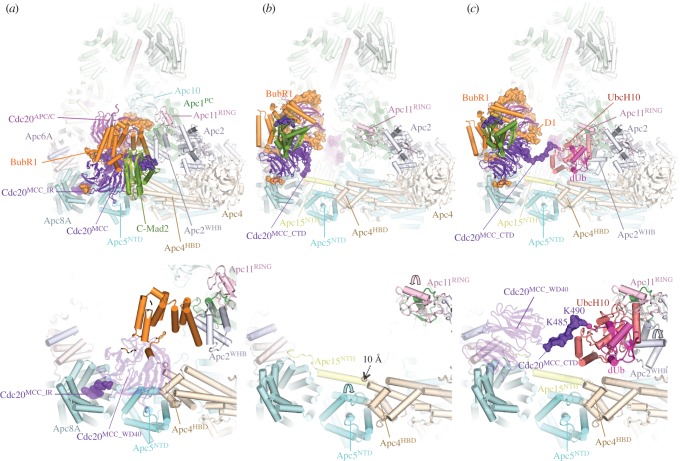
APC/C^MCC^ adopts open and closed states that allows for reciprocal control by the MCC. (*a*) In APC/C^MCC-closed^, the MCC inhibits both substrate (for example, securin and cyclin B) and UbcH10 recognition. Upper panel: overall APC/C^MCC^ structure. Lower panel: shows how binding of the MCC in APC/C^MCC-closed^ causes an upward movement of Apc5^NTD^ and Apc4^HBD^ (compare with *b*) and concomitant disordering of the N-terminal helix of Apc15 (Apc15^NTH^). (*b*) In APC/C^MCC-open^, the catalytic module is exposed, Apc5^NTD^ rotates, Apc4^HBD^ translates down by 10 Å, and Apc15^NTH^ is ordered. Movements of Apc4^HBD^, Apc5^NTD^ and Apc11^RING^ domain are indicated with arrows. (*c*) In the APC/C^MCC^-UbcH10 complex, APC/C^MCC^ adopts the open conformation with Apc15^NTH^ ordered and UbcH10 docking to its canonical position on Apc11^RING^ and Apc2^WHB^. The C-terminal tail of Cdc20^MCC^ engages the catalytic site of UbcH10 for auto-ubiquitination of Lys485 and Lys490. In APC/C^MCC-closed^, the C-terminal IR tail of Cdc20^MCC^ engages the C-box binding site of Apc8A. From Alfieri *et al.* [92].

Having effectively shut down degron recognition by Cdc20^APC/C^, (although not necessarily Cdc20^MCC^), the MCC also represses APC/C's E3 ligase catalytic activity. In the majority of APC/C^MCC^ molecules (in the Alfieri *et al*. study [92]) APC/C^MCC^ adopts a closed conformation (APC/C^MCC-closed^) whereby MCC, through the TPR domain of BubR1, contacts Apc2^WHB^ (figures 11 and 12*a*). This obstructs the UbcH10 binding site on the catalytic module. APC/C^MCC-closed^ is accompanied by an order-to-disorder transition of the Apc15 N-terminal helix (Apc15^NTH^) due to the binding of Cdc20^MCC^ to the platform region. This induces an upward movement of the Apc4 helix bundle domain (Apc4^HBD^) and its adjacent Apc5 N-terminal domain (Apc5^NTD^), disrupting their contacts to Apc15^NTH^ (figure 12*a*,*b*). Interestingly, in a small population of APC/C^MCC^, the molecule adopts an open state (APC/C^MCC-open^) whereby MCC has rotated away from the catalytic module exposing the UbcH10 binding site on Apc2^WHB^ (figure 12*b*). This large repositioning of the MCC is dependent on the disorder-to-order transition of Apc15^NTH^. On transition from APC/C^MCC-closed^ to APC/C^MCC-open^, Apc15^NTH^ rebinds to Apc5^NTD^. This induces a downward rotation of Apc5^NTD^ and downward translation of Apc4^HBD^, displacing the Cdc20^MCC^ binding site on Apc4^HBD^ by 10 Å. Loss of the Cdc20^MCC^-binding interface on the platform releases the MCC to rotate away from the catalytic site of the APC/C (figure 12*a*,*b*).

In the study of Schulman and colleagues the open APC/C^MCC^ conformation predominates [93]. Whatever the cause of difference in the open-closed ratio between the two studies (possibly due to differences in APC/C^MCC^ reconstitution approaches), open APC/C^MCC^ is associated with an ordered conformation of Apc15^NTH^, suggesting that the order-to-disorder transition of Apc15^NTH^ influences the open–closed transition. In support of this notion, deletion of Apc15 locks all APC/C^MCC^ molecules into the closed state, consequently repressing Cdc20^MCC^ auto-ubiquitination [92,93].

The open APC/C^MCC^ conformation would suggest that UbcH10 has the capacity to interact with the APC/C^MCC^, a proposal indeed verified by structures of APC/C^MCC^–UbcH10 complexes (figure 12*c*) [92,93]. In these complexes, UbcH10 is bound to the Apc2^WHB^-Apc11^RING^ catalytic module, as in previous APC/C–UbcH10 complexes [80,111]. The MCC adopts the open conformation with Apc15^NTH^ ordered. Strikingly, the C-terminus of Cdc20^MCC^ engages the catalytic site of UbcH10 with two Lys residues of Cdc20^MCC^ (K485 and K490) accessible to the catalytic site, and which in the reconstituted APC/C^MCC^ are auto-ubiquitinated in an Apc15- and UbcH10-dependent process [92]. Destabilizing closed APC/C^MCC^, either through disrupting the Apc2^WHB^ interface on BubR1 or by deleting the IR tail of Cdc20^MCC^ (which binds Apc8A in closed APC/C^MCC^), promotes Cdc20^MCC^ auto-ubiquitination, even in the absence of Apc15 [92,93].

**Figure 13.**
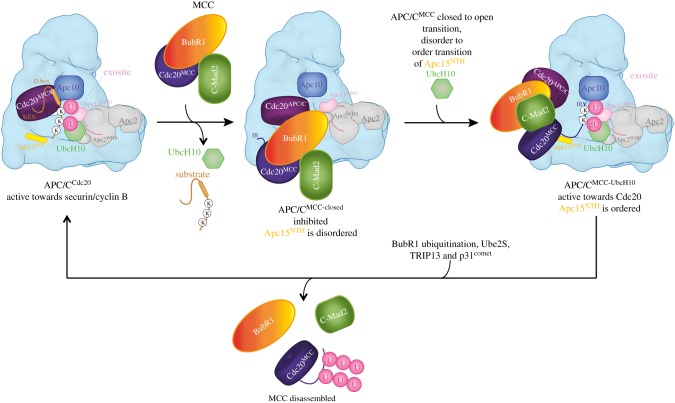
Reciprocal regulation of the APC/C and MCC at the SAC. Cartoon illustrating reciprocal regulation of APC/C and MCC by APC/C^MCC^. MCC binding to APC/C^Cdc20^ generates APC/C^MCC^ that blocks D-box- and KEN-box-dependent substrate binding. APC/C^MCC^ interconverts between APC/C^MCC-closed^ and APC/C^MCC-open^, with APC/C^MCC-closed^ predominating. In APC/C^MCC-open^ the UbcH10 binding site is exposed. UbcH10 binding to APC/C^MCC-open^ catalyses Cdc20^MCC^ auto-ubiquitination. Ube2S elongates Ub-conjugates initiated by UbcH10. Cdc20^MCC^ ubiquitination promotes disassembly of APC/C^MCC^ to generate APC/C^Cdc20^. APC/C^MCC^ disassembly may also be mediated by BubR1 ubiquitination. p31^comet^ and TRIP13 participate in MCC disassembly. During an active SAC, sustained assembly of MCC regenerates APC/C^MCC^. (U: ubiquitin, dU: donor ubiquitin). The position of Bub3 within APC/C^MCC^ is unknown. Adapted from Alfieri *et al.* [92].

These structural and biochemical data show that Cdc20^MCC^ is auto-ubiquitinated by UbcH10 in the context of the open APC/C^MCC^ conformation. This requires Apc15 and thus explains how Apc15 deletion suppresses Cdc20 auto-ubiquitination in a SAC-dependent manner. Because Apc15 deletion blocks progression into anaphase after release from the SAC [59,90,91], it suggests that one mechanism by which the SAC is inactivated is through Cdc20^MCC^ auto-ubiquitination leading to disassembly of APC/C^MCC^, generating active APC/C^Cdc20^ (figure 13). SAC-mediated Cdc20 proteolysis is dependent on the APC/C, Mad2 and BubR1 [94,95,237–240], suggesting that Cdc20^MCC^ ubiquitination occurs in the context of APC/C^MCC^. Consistent with release from mitotic arrest, concomitant with Cdc20 degradation, is the requirement for Apc15 [59,90,91] and Ube2S that would cooperate with UbcH10 to ubiquitinate Cdc20^MCC^, to regulate inactivation of the SAC [173]. *In vitro* Mad2, Cdc20 and BubR1 are released from APC/C^MCC^ following UbcH10-catalysed ubiquitination [91,94,241]. The MCC disassembly products are mainly free BubR1 and Mad2 associated with polyubiquitinated Cdc20. The release of MCC subunits by APC/C^MCC^ was impaired by a lysine free version of Cdc20 [241]. The situation may differ *in vivo* because the Cdc20^K485R/K490R^ mutant did not prevent MCC release from APC/C^MCC^ after a mitotic arrest [59]. However, it is possible that in the K485R/K490R mutant alternative lysines in Cdc20^MCC^ could be ubiquitinated in the context of the APC/C^MCC^, as Schulman and colleagues observed [93].

To ensure efficient MCC release at anaphase onset, additional MCC subunits might also be ubiquitinated. A good candidate is BubR1 that is ubiquitinated in the context of purified APC/C^MCC^ and UbcH10 [241]. Supporting the idea that MCC ubiquitination is required for its release from APC/C^MCC^, depletion of either Apc11 or UbcH10 decreased the amount of MCC dissociated from APC/C^MCC^ after a mitotic arrest [59]. In addition, APC/C regulation of BubR1 homeostasis is essential for correct mitotic timing [242,243]. In APC/C^MCC-open^ the lysine-containing region of BubR1 proximal to D1 is close to the catalytic module of the APC/C, consistent with a model whereby BubR1 is auto-ubiquitinated in the context of APC/C^MCC^ [92] (figures 12*c* and 13).

The architecture and composition of APC/C^MCC^ complexes are conserved in *S. pombe*. However in fission yeast, although Apc15 is also required for Cdc20^MCC^ auto-ubiquitination [232], it additionally functions to exert a checkpoint arrest by stabilizing APC/C–MCC interactions [232,235].

Cdc20^MCC^ auto-ubiquitination is an important event because it contributes to the reciprocal regulation of the APC/C and MCC (figure 13). MCC binding to the APC/C represses APC/C activity. However, in a competing process, dependent on the open conformation of APC/C^MCC^, APC/C^MCC^ is disassembled due to Cdc20^MCC^ auto-ubiquitination [94] and APC/C^Cdc20^ is reactivated. As long as the SAC is active and new MCC is generated, APC/C^MCC^ will reform to suppress APC/C activity. When the SAC is turned off, MCC assembly at the kinetochore stops, and Cdc20^MCC^ auto-ubiquitination allows the spontaneous activation of the APC/C through APC/C^MCC^ disassembly, thereby driving cells into anaphase. This model is consistent with the idea that correctly attached kinetochores do not need to generate and transmit a signal to APC/C^MCC^ to activate the APC/C to initiate chromosome segregation. Only unattached kinetochores signal to regulate the APC/C during the SAC.

*In vivo*, the ratio of the open–closed state of APC/C^MCC^, as well as the factors that influence the open–closed transition and thus Cdc20^MCC^ auto-ubiquitination, are unknown. The SAC arrest protein p31^Comet^, that promotes Cdc20^MCC^ auto-ubiquitination [94,244] (although not *in vitro* [241]), and/or Cdc20 phosphorylation are candidates. Furthermore, USP44 catalyses Cdc20^MCC^ deubiquitination, thereby antagonizing Cdc20^MCC^ ubiquitination to stabilize the APC/C^MCC^, thus sustaining the SAC [95]. Moreover, a ubiquitination-independent pathway functions to disassemble the free MCC through the activities of the AAA^+^ ATPase TRIP13 [245–247], in conjunction with the SAC antagonist p31^Comet^ [248], a C-Mad2 binding protein [249]. p31^Comet^ extracts Mad2 from the MCC [250] and targets C-Mad2-Cdc20 to TRIP13 [251], and competes with BubR1 for C-Mad2 [252].

Errors in controlling accurate chromosome segregation due to defects in the SAC underlie aneuploidy and chromosome instability (CIN), and cause tumour heterogeneity and drug resistance [253]. However, extreme CIN correlates with improved cancer outcome, possibly because karyotypic diversity is required to adapt to selection pressures. A study from Swanton and colleagues found that partial APC/C dysfunction caused by somatic mutations in cancer cell lines lengthened mitosis, suppressed pharmacologically induced chromosome segregation errors and reduced naturally occurring lagging chromosomes. APC/C impairment caused adaptation to Mps1 inhibitors, suggesting a likely resistance mechanism to therapies targeting the SAC [254].

## 9. Spatial regulation of the APC/C

Specifics of the intracellular location and spatial control of the APC/C are relatively little understood [255]. The APC/C is localized to centrosomes through Emi1–NUMA complexes [256], the recently identified KIAA1430 protein [257] and to the chromosomes through interactions mediated by the Ska3 complex [258,259].

## 10. Conclusion and perspectives

An enduring question asked about the APC/C is why is it so large? The simplest response must be that its size reflects its central role in coordinating critical transitions during the cell cycle. This requires control at multiple levels (through interchangeable coactivator subunits, phosphorylation, the SAC and Emi1, often exerted through allosteric conformational changes of the APC/C), and the capacity of the APC/C to change its substrate specificity during different phases of the cell cycle, transition through which is controlled by the APC/C. Thus, the APC/C possesses intrinsic self-control mechanisms. This is probably best exemplified by the auto-ubiquitination of Cdc20^MCC^ that enables spontaneous activation of the APC/C at anaphase only when all chromosomes have achieved correct bipolar attachment to the mitotic spindle. The large size of the APC/C is contributed by the seven scaffolding proteins, four of which form structurally related homo-dimers, which stack in parallel to create the pseudo-dyad symmetric TPR lobe. This TPR lobe, together with Apc1, Apc4 and Apc5 of the platform, assembles the scaffold to juxtaposition the catalytic and substrate recognition modules. The structural symmetry of the TPR lobe generates multiple structurally related binding sites that engage the C box and IR tail of coactivators and IR tail of Apc10. The structural equivalence of the C-box binding site of Apc8B and the IR-tail binding sites of Apc3 is illustrated by the engagement of the Cdc20^MCC^ IR tail with the C-box binding site of Apc8A [92,93]. The APC/C is a distant ancestor of the large CRL family of E3 ligases. Both share a conserved catalytic module of RING and cullin subunits. How the APC/C evolved from the simpler CRLs is not clear because there are no obvious intermediate complexes. All the large scaffolding subunits comprise multiple repeat motif domains. TPR and WD40 repeat motifs are ubiquitous in proteins involved in protein–protein interactions, and it is intriguing that the only other known instances of the PC repeat domain are the Rpn1 and Rpn2 subunits of the 19S regulatory particle of the proteasome [85].

Of interest is the realization that the APC/C functions by engaging coactivators, substrates and inhibitors through recognition of short linear sequence motifs, for example degrons, C box, IR tail and MR tail [120]. Conformational variability of small domains attached to the APC/C scaffold through flexible linkers (Apc2^WHB^, Apc11^RING^ and the WD40 domains of coactivators) has important implications for mediating catalysis and regulation.

Despite the huge progress in understanding the function and mechanism of the APC/C during the two decades since its discovery, much needs to be explored. We still have little molecular understanding of how the APC/C selects different substrates during the cell cycle. This will require cryo-EM structures of different states of the APC/C in complex with full-length intact substrates, in addition to more quantitative determinations of the affinities of different APC/C complexes for their cognate substrates. To what extent APC/C–substrate affinities compared with the catalytic efficiency of lysine ubiquitination (determined by substrate–lysine proximity to the E2 catalytic site and the competing rates of deubiquitination) controls the rate of substrate degradation is unclear. The level to which the intracellular location of APC/C complexes controls their various functions and how this is subject to cell-cycle regulation are also still largely unexplored.

## Data accessibility

This article has no additional data.
